# Gut environment-induced intraepithelial autoreactive CD4^+^ T cells suppress central nervous system autoimmunity via LAG-3

**DOI:** 10.1038/ncomms11639

**Published:** 2016-05-20

**Authors:** Atsushi Kadowaki, Sachiko Miyake, Ryoko Saga, Asako Chiba, Hideki Mochizuki, Takashi Yamamura

**Affiliations:** 1 Department of Immunology, National Institute of Neuroscience, National Center of Neurology and Psychiatry, 4-1-1 Ogawahigashi, Tokyo 187-8502, Japan; 2 Department of Neurology, Osaka University Graduate School of Medicine, 2-2 Yamadaoka, Suita 565-0871, Japan; 3 Department of Immunology, Juntendo University School of Medicine, 2-1-1 Hongo, Tokyo 113-8421, Japan

## Abstract

The gut environment has been found to significantly influence autoimmune diseases such as multiple sclerosis; however, immune cell mechanisms are unclear. Here we show that the gut epithelium of myelin oligodendrocyte glycoprotein(35-55)-specific T-cell receptor transgenic mice contains environmental stimuli-induced intraepithelial lymphocytes (IELs) that inhibit experimental autoimmune encephalomyelitis on transfer. These cells express surface markers phenotypical of ‘induced' IELs, have a T_H_17-like profile and infiltrate the central nervous system (CNS). They constitutively express *Ctla4* and *Tgfb1* and markedly upregulate *Lag3* expression in the CNS, thereby inhibiting inflammation. We also demonstrate the suppressive capability of CD4^+^ IELs with alternative antigen specificities, their proliferation in response to gut-derived antigens and contribution of the microbiota and dietary aryl hydrocarbon receptor ligands to their induction. Thus, the gut environment favours the generation of autoreactive CD4^+^ T cells with unique regulatory functions, potentially important for preventing CNS autoimmunity.

## Abstract (web summary)

Experimental autoimmune encephalomyelitis (EAE) involves inflammatory cell infiltration into the central nervous system (CNS) and models the human disease multiple sclerosis. Here the authors show that transferred CD4+ gut intraepithelial lymphocytes can migrate into the CNS and inhibit inflammation in recipient mice with EAE.

## Graphical abstract

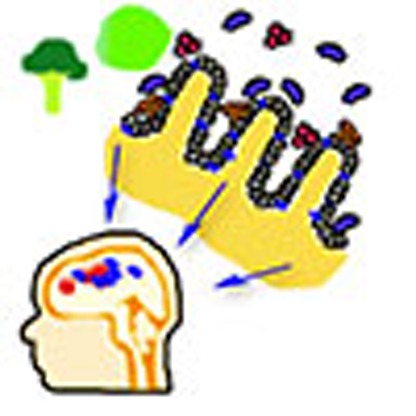

Growing evidence supports the hypothesis that an altered balance between pathogenic interleukin (IL)-17^+^ or interferon gamma (IFNγ)^+^ helper T (T_H_) cells and immunoregulatory cells with anti-inflammatory potential and the subsequent breakdown of immune tolerance may underlie the pathogenesis of autoimmune diseases, including multiple sclerosis (MS)^1^. Although cellular and molecular mechanisms involved in the generation or attenuation of potentially pathogenic autoreactive T_H_ cells remain unclear, the gut mucosa, the largest immune organ that interacts with the external environment, is a possible location for the generation of effector T cells that cause autoimmune responses^2^,^3^,^4^ and regulatory T cells that prevent these responses^5^,^6^,^7^.

Changes in the gut environment can lead to alterations of experimental autoimmune encephalomyelitis (EAE), a rodent model of MS^2^,^4^,^7^,^8^. MS is an autoimmune disease that causes myelin destruction in the central nervous system (CNS). Epidemiological data indicate that both genetic and environmental factors are involved in MS pathogenesis. Although genome-wide association studies indicate that single-nucleotide polymorphisms (SNPs) of key molecules in T_H_ cell differentiation pathways are linked to MS susceptibility^9^,^10^, an increase in the number of MS patients in developed countries, including Japan, might be attributable to environmental changes^11^,^12^,^13^. We previously reported that an oral antibiotic treatment that altered the gut flora could decrease EAE severity^8^. Subsequently, clinical manifestations of actively induced EAE or spontaneous EAE in TCR-transgenic mice were shown to be attenuated in germ-free (GF) mice^2^,^3^. Recolonizing GF mice with a full complement of commensal bacteria or even with segmented filamentous bacteria alone restored gut T_H_17 cells in mice, along with the ability of the mice to develop EAE^3^. In contrast, clostridial strains or *Bacteroides fragilis* polysaccharide A induced Foxp3^+^ regulatory T cells that could regulate the colitis and CNS inflammation that accompanies EAE^5^,^6^,^7^. However, inflammatory T_H_17 cells can acquire a regulatory phenotype after being recruited into the small intestine, as demonstrated in a model of systemic tolerance induced by anti-CD3 antibody^14^. Dietary fatty acids also influence gut T-cell differentiation and EAE disease course^4^. Therefore, the gut and gut-associated lymphoid system are probable sites for functional maturation of autoimmune pathogenic T cells and regulatory T cells capable of suppressing autoimmune inflammation outside the gut.

Myelin oligodendrocyte glycoprotein (MOG)-specific T-cell receptor (TCR) transgenic (2D2) mice^15^ are often used to study MS pathogenesis, as a proportion of these mice spontaneously develop EAE several months after birth. Although precise mechanisms are not fully understood, pathogenesis in 2D2 mice may depend on the balance between monoclonal T cells with pathogenic potential and those with regulatory functions. Here we use this model to investigate how gut-resident T cells might play a role in CNS autoimmune disease.

First, we reveal that two distinct populations of T cells expressing MOG-specific TCR (2D2-TCR) are abundant in the small intestinal epithelium of 2D2 mice. These cells have either low or high expression of 2D2-TCR and a phenotype of CD2^−^CD5^−^ ‘natural' intraepithelial lymphocytes (IELs) or CD2^+^CD5^+^ ‘induced' IELs, according to the definition by Cheroutre *et al*.^16^ Natural IELs acquire an activated phenotype during thymic development in the presence of self-antigens, whereas induced IELs are derived from conventional T cells and acquire their activated phenotype and functionally differentiate post-thymically in response to cognate antigens. It remains unclear whether they have the potential to regulate autoimmune diseases in extraintestinal organs.

Here we demonstrate that adoptive transfer of CD4^+^ induced IELs from 2D2 mice to wild-type (WT) mice with MOG(35-55)-induced EAE ameliorates disease severity, although transfer of other IEL populations does not. CD4^+^ induced IELs efficiently migrate into the CNS and upregulate critical immune regulatory molecules, such as LAG-3. CD4^+^ induced IELs inhibit T-cell proliferation *in vitro* by a mechanism dependent on LAG-3, CTLA-4 and transforming growth factor-beta (TGF-β). We show the inhibitory capacity of CD4^+^ induced IELs with another TCR associated with arthritis and with the polyclonal TCR of WT mice. The autoreactive CD4^+^ induced IELs proliferate in response to gut-derived antigens. Finally, we demonstrate that gut environmental stimuli, such as the microbiota and aryl hydrocarbon receptor (AHR) ligands in certain diets, contribute to CD4^+^ IEL induction. These findings indicate that the gut environment can favour the generation of autoreactive CD4^+^ T cells with unique regulatory functions. Such regulatory cells may have an important role in controlling extraintestinal autoimmune diseases, such as MS.

## Results

### Characterization of IELs in 2D2 mice

**Figure 1:**
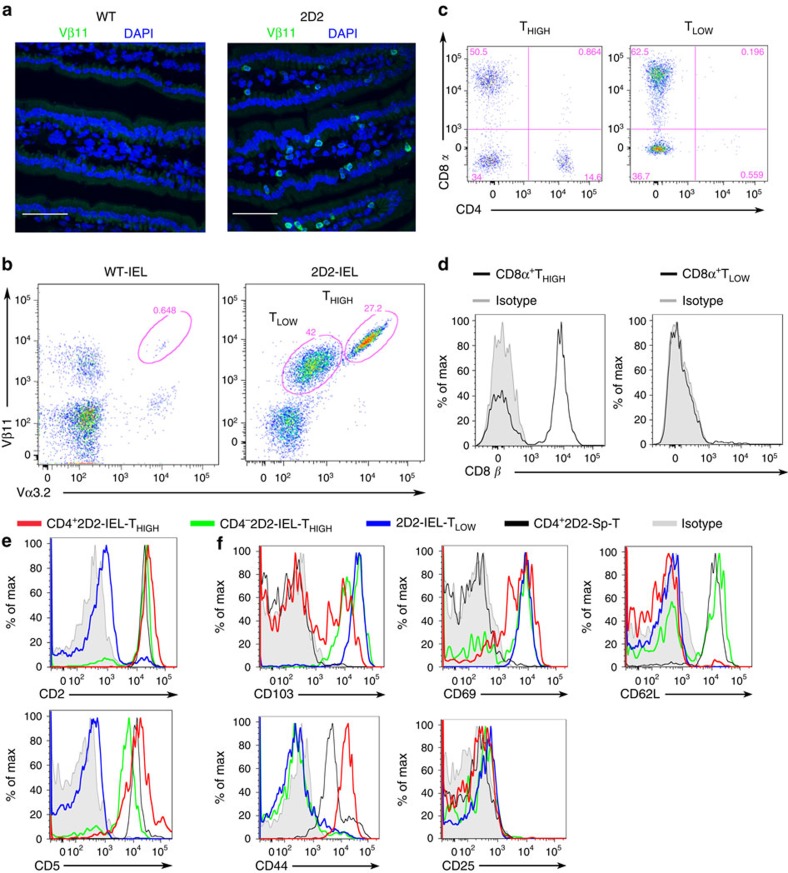
IELs in 2D2 mice. (**a**) Immunohistochemistry of the small intestines of WT and 2D2 mice. Cryosections were stained with anti-Vβ11 Ab (green) and nuclear DNA-staining DAPI (blue). Original magnification: × 600. Scale bars, 50 μm. Images shown are representative of two experiments. (**b**) Expression of Vα3.2^+^Vβ11^+^ TCR (2D2-TCR) in TCRβ^+^ IELs of WT or 2D2 mice was assessed by flow cytometry. 2D2-mouse IEL T cells with high expression of 2D2-TCR (2D2-IEL-T_HIGH_) are indicated as T_HIGH_, and those with low expression of 2D2-TCR (2D2-IEL-T_LOW_) are labelled as T_LOW_. (**c**) Expression of co-receptors (CD4 and CD8α) on 2D2-IEL T cells. (**d**) Expression of CD8β on CD8α^+^ T cells among 2D2-IEL-T_HIGH_ and -T_LOW_ cells. (**e**) Expression of CD2 and CD5 on 2D2-IEL cells and 2D2-spleen CD4^+^ T cells (CD4^+^2D2-Sp-T). 2D2-IEL populations were analysed and grouped as CD4^+^2D2-IEL-T_HIGH_, CD4^−^-2D2-IEL-T_HIGH_ and 2D2-IEL-T_LOW_ cells. (**f**) Expression of CD103 and T-cell activation markers on 2D2-IEL cells and 2D2-spleen CD4^+^ T cells. (**b**–**f**) Data are representative of more than three experiments. DAPI, 4,6-diamidino-2-phenylindole.

Our initial question was whether MOG(35-55)-reactive TCR-transgenic mice (2D2) would harbour IELs in the intestinal mucosa, even though the large majority of CD4^+^ T cells express Vα3.2 and Vβ11 TCR chains (2D2-TCR) (Supplementary Fig. 1). Despite the limited TCR specificity, the epithelium of the small intestine was inhabited by abundant Vβ11^+^ T cells, corresponding to 2D2 IELs (Fig. 1a). Signs of pathological inflammation or altered architecture of the gut mucosa were not observed by histology (Supplementary Fig. 2). We next analysed the phenotype of small intestinal IELs by flow cytometry. Interestingly, the IELs were divided into two distinct T-cell populations with high or comparatively low 2D2-TCR expression: 2D2-IEL-T_HIGH_ and 2D2-IEL-T_LOW_ (Fig. 1b).

The 2D2-IEL-T_HIGH_ cells comprised CD8α^+^, CD4^+^ and double-negative (DN) populations, whereas the 2D2-IEL-T_LOW_ cells were CD8α^+^ or DN (Fig. 1c). The majority of CD8α^+^ cells in the T_HIGH_ cell population were CD8β^+^, suggesting that they are CD8αβ^+^ T cells. In contrast, almost all of the CD8 α^+^ cells in the T_LOW_ group were CD8β^−^ and thought to represent CD8αα^+^ T cells (Fig. 1d). Regardless of CD4 expression, T_HIGH_ cells were positive for CD2 and CD5 (Fig. 1e). In this regard, they resembled induced IELs derived from WT mice^16^. In contrast, T_LOW_ cells were negative for CD2 and CD5, similar to natural IELs of WT mice. We further examined the expression of surface markers in the 2D2-IEL population. CD4^+^2D2-IEL-T_HIGH_ cells comprised both CD103^+^ and CD103^−^ cells and had a CD69^+^CD62L^−^CD44^+^ phenotype that is shared by WT-CD4^+^ induced IELs (Fig. 1f and Supplementary Fig. 3a). Although this phenotype is a marker of activated T cells, the CD4^+^2D2-IEL-T_HIGH_ cells are thought to be partially, but not fully, activated, because they hardly express CD25 (% CD25, mean±s.e.m.: 0.31±0.00043; Fig. 1f). The CD4^−^2D2-IEL-T_HIGH_ cells resembled WT-CD8^+^ induced IEL, as they were CD103^+^, CD69^+^, CD62L^+/−^, CD44^−^ and CD25^−^. However, a comparatively high level of CD62L expression and low level of CD44 expression were observed in CD4^−^2D2-IEL-T_HIGH_ cells (Fig. 1f and Supplementary Fig. 3b). 2D2-IEL-T_LOW_ cells were CD103^+^, CD69^+^, CD62L^−^, CD44^low/−^ and CD25^−^, similar to WT natural IELs (Fig. 1f and Supplementary Fig. 3b).

**Figure 2:**
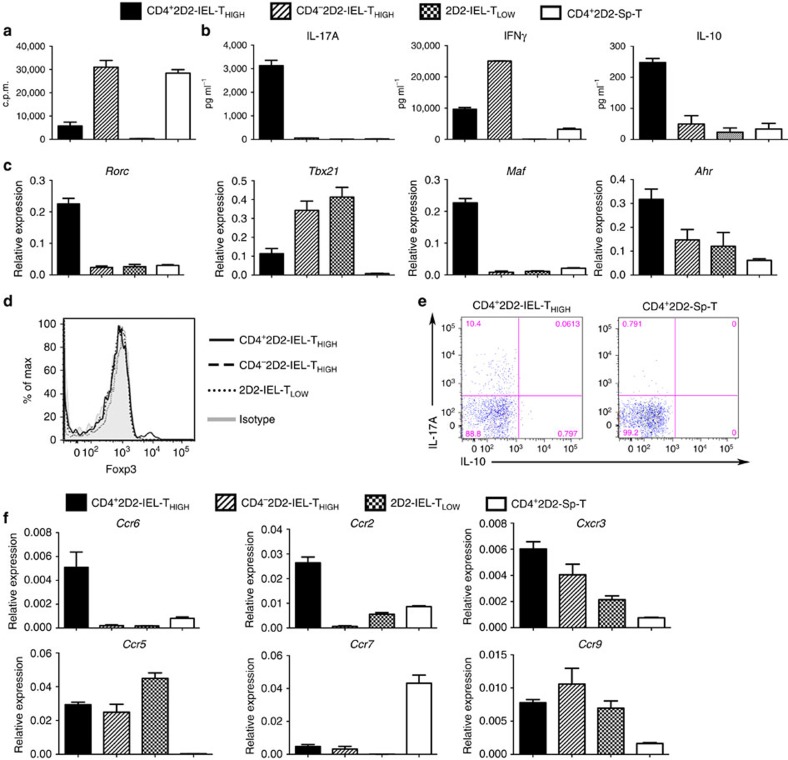
Functional characterization of 2D2-IEL populations. (**a**) Sorted populations of 2D2-IEL (CD4^+^ and CD4^−^, 2D2-IEL-T_HIGH_ and 2D2-IEL-T_LOW_) or spleen CD4^+^ T (CD4^+^2D2-Sp-T) cells from 2D2 mice were stimulated with anti-CD3 and anti-CD28 mAb for 3 days *in vitro.* Cell proliferation was measured by incorporation of [^3^H] thymidine (c.p.m.) (data represent mean and s.e.m. of triplicates). (**b**) Supernatants from cultures used in **a** were analysed for cytokine production (mean±s.e.m. of triplicates). Representative data of two experiments are shown in **a** and **b**. (**c**) Gene expression of the indicated transcription factors. mRNA obtained from 2D2-IEL (CD4^+^- or CD4^−^-T_HIGH_ and -T_LOW_) or 2D2-spleen-CD4^+^ T cells was analysed by quantitative RT–PCR (*n*=3) (mean and s.e.m.). The expression levels were normalized to that of *Gapdh* (glyceraldehyde phosphate dehydrogenase). (**d**) Intracellular Foxp3 staining of 2D2-IEL and 2D2-spleen CD4^+^ T cells. (**e**) Intracellular staining of IL-17A and IL-10 in CD4^+^2D2-IEL-T_HIGH_ cells and 2D2-spleen-CD4^+^ T cells. Cells were stimulated with PMA (500 ng ml^−1^) plus ionomycin (500 ng ml^−1^) (**d**,**e**). Data are representative of two experiments. (**f**) Expression of chemokine receptors analysed by quantitative RT–PCR as in **c** (mean and s.e.m.). c.p.m., counts per minute; PMA, phorbol 12-myristate 13-acetate; RT–PCR, reverse transcription PCR.

To characterize the 2D2-IEL populations further, we examined their proliferation and cytokine production on stimulation with anti-CD3 and anti-CD28 monoclonal antibodies (mAbs) (Fig. 2a,b). The proliferative responses of CD4^+^2D2-IEL-T_HIGH_ cells were lower than those of splenic CD4^+^2D2-T cells, and 2D2-IEL-T_LOW_ cells did not respond to anti-CD3/anti-CD28 stimulation, indicating that they are similar to WT-CD4^+^ induced and natural IELs in their responsiveness to TCR stimulation^17^ (Fig. 2a). CD4^+^2D2-IEL-T_HIGH_ cells produced substantial amounts of the inflammatory cytokines IL-17A and IFNγ and anti-inflammatory cytokine IL-10 (Fig. 2b). However, CD4^−^2D2-IEL-T_HIGH_ cells produced IFNγ, but did not produce IL-17A. Cytokine production by 2D2-IEL-T_LOW_ cells was very low. To compare these phenotypically different 2D2-IEL populations further, we examined mRNA expression of the transcription factors involved in T_H_ cell development. Consistent with their cytokine production, the CD4^+^2D2-IEL-T_HIGH_ cells expressed *Rorc* and *Tbx21* (Fig. 2c). They also expressed *Maf* and *Ahr*, which were previously shown to be expressed by non-pathogenic T_H_17 or Tr1 cells^18^,^19^. The CD4^−^2D2-IEL-T_HIGH_ cells and 2D2-IEL-T_LOW_ cells expressed *Tbx21*. Foxp3 expression in the CD4^+^2D2-IEL-T_HIGH_ cells was restricted to a very small population (% Foxp3, mean±s.e.m.: 3.51±0.30), and almost all of the Foxp3^+^ cells were CD25 negative (Fig. 1f) and lacked regulatory functions^20^. All other IEL populations were Foxp3 negative (Fig. 2d). Almost none of the IL-17A-producing cells in the CD4^+^2D2-IEL-T_HIGH_ population co-produced IL-10, which distinguishes them from previously reported regulatory T_H_17 cells (Fig. 2e) (refs 14, 21). *Ccr6*, *Ccr2*, *Cxcr3* and *Ccr5* were highly expressed in the CD4^+^2D2-IEL-T_HIGH_ cells (Fig. 2f). The expression of *Ccr7* was low and gut-tropic chemokine receptor *Ccr9* expression was high in all IEL populations (Fig. 2f). These data collectively indicate that the 2D2-IEL-T_HIGH_ and 2D2-IEL-T_LOW_ cells corresponded to induced IELs and natural IELs, respectively.

### Adoptively transferred CD4^+^2D2-IEL-T_HIGH_ cells improve EAE

**Figure 3:**
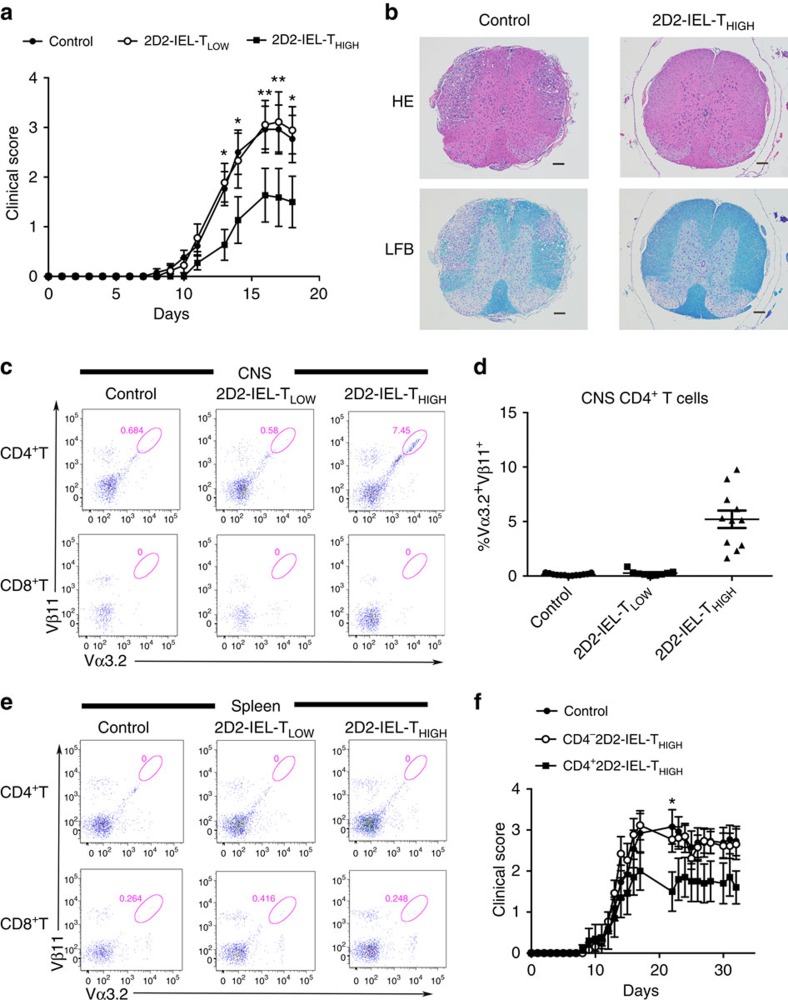
Suppression of EAE by adoptive transfer of 2D2-IEL-T_HIGH_ cells. (**a**) In total, 5.0 × 10^5^ WT spleen cells (control) and 2D2-IEL-T_HIGH_ or -T_LOW_ cells were transferred intraperitoneally (day −1), and EAE was induced on the following day (day 0). Clinical scores of EAE were assessed (mean and s.e.m.). (**b**) Representative H&E and LFB staining of spinal cords from control recipients (*n*=4) or 2D2-IEL-T_HIGH_ cell recipients (*n*=3) 3 weeks after EAE induction. Scale bars, 100 μm. (**c**) Representative flow cytometry analysis of 2D2-TCR (Vα3.2^+^Vβ11^+^) expression in CD4^+^ or CD8^+^ T cells obtained from the CNS of EAE mice after peak disease. (**d**) The total proportion (%) of 2D2-TCR^+^ cells among the CNS-infiltrating CD4^+^ T cells obtained from (**c**). Plots represent data from individual animals (mean and s.e.m.). Data in **a**,**c** and **d** were pooled from two experiments where 13 control, 9 2D2-IEL-T_LOW_ and 11 2D2-IEL-T_HIGH_ mice were induced for EAE. (**e**) When CNS cells were separated for analysis (**d**), spleen cells from the mice were also obtained, and proportions of Vα3.2^+^Vβ11^+^ 2D2 T cells in CD4^+^ or CD8^+^ T cells among these cells were analysed by FACS. Spleen cells from four mice were pooled from each group. Data are representative of two experiments. (**f**) In total, 2.0 × 10^5^ WT spleen cells (control) or CD4^−^ or CD4^+^2D2-IEL-T_HIGH_ cells were adoptively transferred intravenously (day −1) and EAE induced on the following day (day 0). Clinical scores of mice receiving control (*n*=13), CD4^−^2D2-IEL-T_HIGH_ (*n*=13) and CD4^+^2D2-IEL-T_HIGH_ (*n*=10) cells were pooled from two experiments (mean and s.e.m.). *P<0.05, **P<0.01 by two-way analysis of variance with Bonferroni's post-test. FACS, fluorescence-activated cell sorting; H&E, haematoxylin and eosin; LFB, Luxol fast blue.

Accumulating evidence indicates that IELs play both inductive and protective roles for local intestinal inflammation^16^. To clarify the roles of IELs in regulating autoimmune inflammation in extraintestinal organs, we explored the effects of transferring 2D2-IEL populations into EAE mice. We adoptively transferred 2D2-IEL-T_HIGH_, 2D2-IEL-T_LOW_ or WT control spleen cells into WT recipient mice and induced EAE by immunization with MOG(35-55) peptide. Transfer of 2D2-IEL-T_HIGH_ cells significantly reduced the severity of EAE, although transfer of 2D2-IEL-T_LOW_ cells did not show significant effects (Fig. 3a). Moreover, histological examination of the spinal cord 3 weeks after adoptive transfer showed less mononuclear cell infiltration and lower grades of demyelination in mice treated with 2D2-IEL-T_HIGH_ cells as compared with control mice (Fig. 3b). This result was consistent with the reduced disease severity in 2D2-IEL-T_HIGH_ cell-treated mice shortly before they were killed (EAE score, mean±s.e.m.: control, 2.8±0.48; 2D2-IEL-T_HIGH_, 0.33±0.33; *P*<0.05 by unpaired *t*-test).

CD4^+^ T cells, but not CD8^+^ or DN T cells, expressing 2D2-TCR (Vα3.2^+^Vβ11^+^) were present in the CNS of recipient mice that received 2D2-IEL-T_HIGH_ cells (Fig. 3c,d and Supplementary Fig. 4a). However, CD4^+^2D2-TCR^+^ cells were not detected in the spleens of the recipient mice (Fig. 3e). These results indicated that CD4^+^2D2-IEL-T_HIGH_ cells could efficiently infiltrate inflamed CNS tissues and participate in the on-going inflammatory process, possibly as immunoregulatory lymphocytes.

We next transferred CD4^+^ or CD4^−^2D2-IEL-T_HIGH_ cells to WT recipients and immunized the mice with MOG(35-55). We confirmed that the transfer of CD4^+^2D2-IEL-T_HIGH_ cells ameliorated clinical EAE (Fig. 3f), and that the transferred T cells were detected in the CNS of the recipients (Supplementary Fig. 4b). In contrast, transfer of CD4^−^2D2-IEL-T_HIGH_ cells showed no significant effect on EAE development (Fig. 3f). We transferred 2D2-IEL-T_HIGH_ cells to congenic EAE mice and confirmed unequivocally that the Vα3.2^+^Vβ11^+^ cells in the recipients' CNS were the transferred cells (Supplementary Fig. 5). These data suggest that CD4^+^2D2-IEL-T_HIGH_ cells could specifically traffic to inflammatory lesions in the CNS and inhibit EAE.

### 2D2-IEL-T_HIGH_ cells suppress peripheral T_H_17 induction

**Figure 4:**
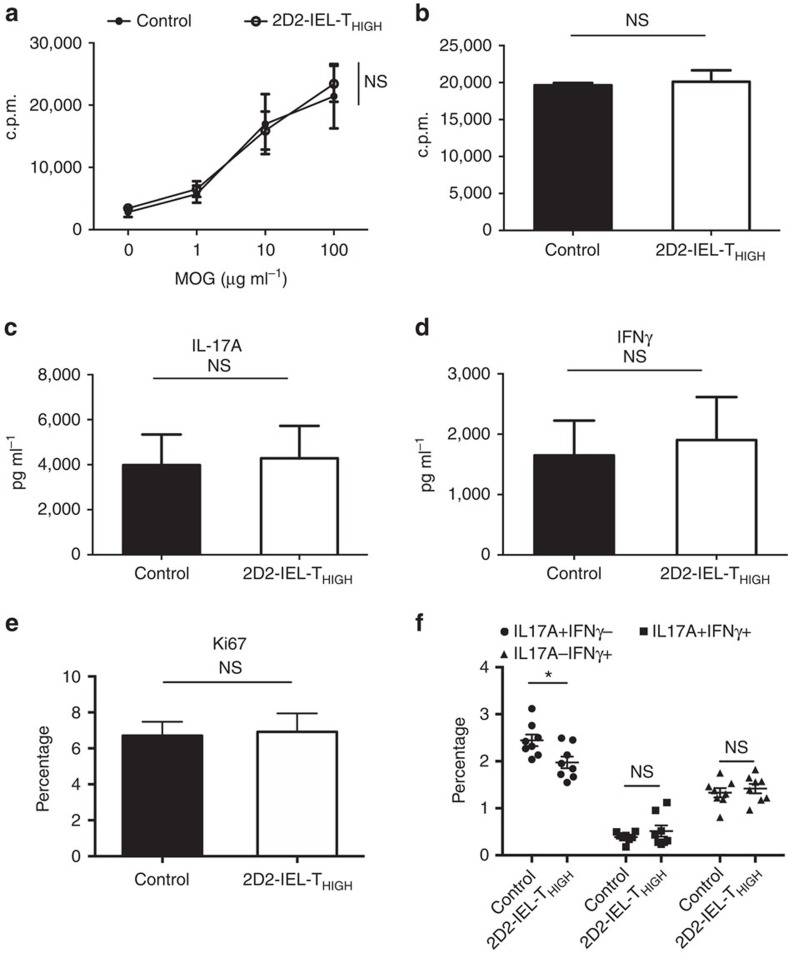
The effect of 2D2-IEL-T_HIGH_ cells on the peripheral activation of CD4^+^ T cells. (**a**) In total, 5.0 × 10^5^ 2D2-IEL-T_HIGH_ cells or WT spleen cells were adoptively transferred intraperitoneally to WT recipients (day −1) that were immunized with MOG(35-55) on the following day (day 0) without pertussis toxin (*n*=4 per group). On day 10, inguinal lymph node cells were obtained and cultured with the indicated doses of MOG(35-55) or anti-CD3 mAb. (**b**) Cells were treated with [^3^H]thymidine for the proliferation assay (c.p.m.). (**c**,**d**) In parallel with experiment **a**, supernatants of cells stimulated with 100 μg ml^−1^ of MOG(35-55) were collected and analysed for cytokine production. Data are representative of three experiments (mean and s.e.m.). (**e**,**f**) 2D2-IEL-T_HIGH_ cells or WT-spleen cells were adoptively transferred, and recipient mice immunized with MOG(35-55) as in **a**. On day 11, inguinal lymph node cells were collected and stained for intracellular Ki67 (**e**) or stimulated with PMA (50 ng ml^−1^) plus ionomycin (500 ng ml^−1^) and stained for IL-17A and IFNγ (**f**). (**e**,**f**) Percentages in CD4^+^ T cells are displayed (mean and s.e.m.). Plots in (**f**) represent data from individual animals. *n*=8 per group, pooled from two experiments. (**a**,**f**) NS (not significant) or *P<0.05 by two-way analysis of variance with Bonferroni's post-test. (**b**–**e**) NS (not significant) by unpaired *t*-test. c.p.m., counts per minute; PMA, phorbol 12-myristate 13-acetate.

To better understand the mechanism by which transfer of 2D2-IEL-T_HIGH_ cells ameliorates the development of EAE, we next examined the proliferative responses and cytokine production of draining lymph node (dLN) cells at the site of immunization using MOG(35-55)-immunized WT mice that received either 2D2-IEL-T_HIGH_ cells or control cells (WT spleen cells). First, we tested this by *in vitro* cell culture experiments. There was no difference in the proliferative response to MOG(35-55) (Fig. 4a) or to anti-CD3 mAb (Fig. 4b) between the control and 2D2-IEL-T_HIGH_ recipient mice. Production of IL-17A and IFNγ following MOG(35-55) stimulation was also comparable between both groups of mice (Fig. 4c,d). Next, we measured the expression levels of the cellular proliferation marker Ki67 and production of IL-17/IFNγ in dLN-CD4^+^ T cells *ex vivo*. There was no difference in Ki67 expression between the control and 2D2-IEL-T_HIGH_ recipient mice (Fig. 4e). However, the proportion of IL-17A^+^IFNγ^−^ cells was slightly but significantly reduced, whereas the proportion of IL-17A^+^IFNγ^+^ and IL-17A^−^IFNγ^+^ cells was not altered (Fig. 4f). These results indicate that the adoptive transfer of 2D2-IEL-T_HIGH_ cells has an effect on peripheral T_H_17 differentiation during EAE. However, the effect was slight, and we assume that the transferred CD4^+^2D2-IEL-T_HIGH_ cells directly regulate inflammation in the CNS.

### CD4^+^2D2-IEL-T_HIGH_ cells express regulatory genes in the CNS

**Figure 5:**
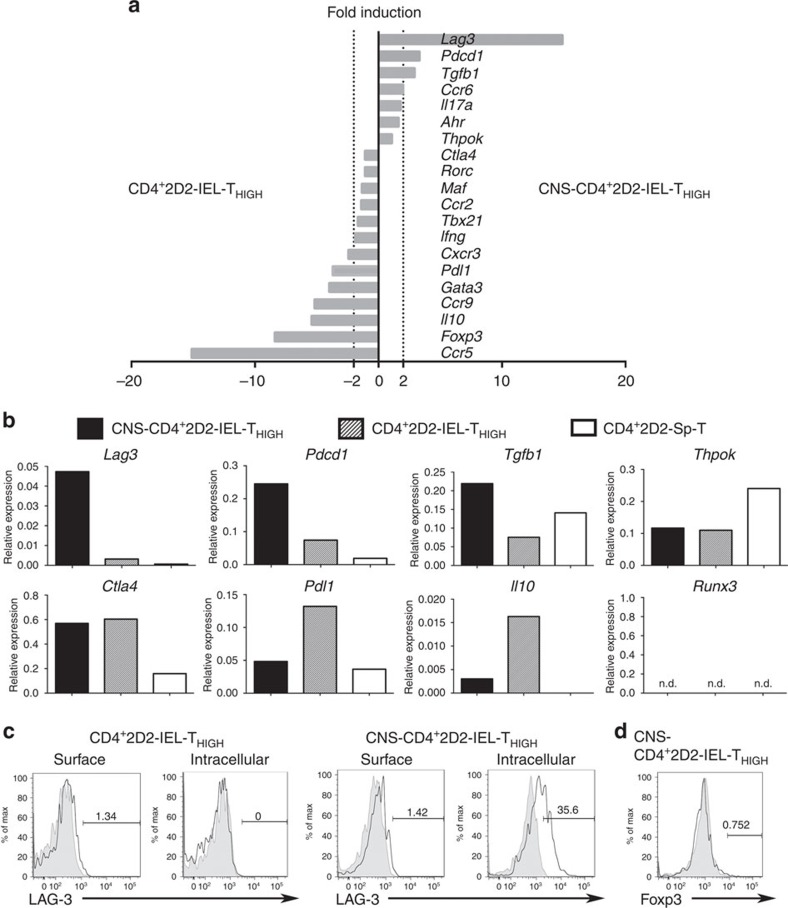
Comparison of gut-resident and CNS-infiltrating CD4^+^2D2-IEL-T_HIGH_ cells. (**a**) CD4^+^2D2-IEL-T_HIGH_ cells were sorted from the IELs of 2D2 mice. 2D2-IEL-T_HIGH_ cells (5.0 × 10^5^) were adoptively transferred to WT mice (day −1) and EAE was induced (day 0). Eighteen or twenty days later, CD4^+^2D2-TCR^+^ cells were recovered from the CNS of WT recipients (CNS CD4^+^2D2-IEL-T_HIGH_). Spleen CD4^+^2D2-T cells were also sorted for comparison (Sp-T). mRNA was obtained from each sample, and quantitative RT–PCR of each indicated gene was performed. Gene expression levels were normalized to that of *Gapdh*. Fold-induction of the indicated gene expression in CD4^+^2D2-IEL-T_HIGH_ and CNS-CD4^+^2D2-IEL-T_HIGH_ cells was compared. (**b**) The expression levels of selected genes in CD4^+^2D2-IEL-T_HIGH_, CNS-CD4^+^2D2-IEL-T_HIGH_ and SpCD4T cells were compared. Each sample was pooled from four mice. The mean expression of duplicate samples was calculated. (**c**) In total, 2.0 × 10^6^ 2D2-IEL-T_HIGH_ cells were transferred (day −1) to WT recipients and EAE was induced (day 0). Nineteen days later, the surface and intracellular expression of LAG-3 in CD4^+^2D2-IEL-T_HIGH_ (left) or the CNS-CD4^+^2D2-IEL-T_HIGH_ (right) cells were assessed. (**d**) In total, 7.5 × 10^5^ 2D2-IEL-T_HIGH_ cells were transferred to WT recipients (day −1) and EAE was induced (day 0). Eighteen days later, Foxp3 expression in CNS-CD4^+^2D2-IEL-T_HIGH_ cells was analysed. Data are representative of three (**c**) or two experiments (**d**). RT–PCR, reverse transcription PCR.

We next compared the gene expression profile of CD4^+^2D2-IEL-T_HIGH_ cells obtained from the gut of 2D2 mice and that of CNS-infiltrating cells (CNS-CD4^+^2D2-IEL-T_HIGH_) isolated from WT EAE mice that received 2D2-IEL-T_HIGH_ cells (Fig. 5a). We examined the expression of genes associated with immune regulation, cytokines, transcription factors and chemokine receptors, whose roles in the development or function of T_H_ cells have been previously documented. Compared with gut-derived CD4^+^2D2-IEL-T_HIGH_ cells, the CNS-CD4^+^2D2-IEL-T_HIGH_ cells were characterized by lower expression levels of T_H_1-associated genes (*Ccr5*, *Cxcr3* and *Ifng*) and the T_H_2-associated gene *Gata3*. Downregulation of *Ccr9* was also noted in the CNS-CD4^+^2D2-IEL-T_HIGH_ cells. The CNS-CD4^+^2D2-IEL-T_HIGH_ and gut-derived CD4^+^2D2-IEL-T_HIGH_ cells expressed similar levels of T_H_17-associated genes, including *Maf*, *Rorc*, *Ahr* and *Il17a*, and a similar level of *Ccr2* (Fig. 5a). However, CNS-CD4^+^2D2-IEL-T_HIGH_ cells showed higher expression levels of *Ccr6* than gut-derived CD4^+^2D2-IEL-T_HIGH_ cells. Mutual expression of *Thpok* and *Runx3* is reported to regulate intestinal CD4^+^ T-cell function^22^. CD4^+^2D2-IEL-T_HIGH_ cells and CNS-CD4^+^2D2-IEL-T_HIGH_ cells expressed similarly reduced levels of *Thpok*, when compared with CD4^+^ T cells from the spleen of 2D2 mice (Fig. 5a,b). *Runx3* expression was below the detection limit. It is also noteworthy that *Foxp3* expression in the CNS-CD4^+^2D2-IEL-T_HIGH_ cells was lower than that in the gut-derived CD4^+^2D2-IEL-T_HIGH_ cells (Fig. 5a), excluding the possibility of selective recruitment of Foxp3^+^ regulatory T cells in the CNS.

Among the genes related to immune suppression, *Lag3*, *Pdcd1* and *Tgfb1* were upregulated in the CNS-CD4^+^2D2-IEL-T_HIGH_ cells (Fig. 5b). *Ctla4* was similarly upregulated in both the CD4^+^2D2-IEL-T_HIGH_ cells and CNS-CD4^+^2D2-IEL-T_HIGH_ cells. However, the expression of *Pdl1* and *Il10* was lower in the CNS-CD4^+^2D2-IEL-T_HIGH_ cells. Thus, *Lag3*, *Tgfb1* and *Ctla4* are candidate genes for involvement in the CD4^+^2D2-IEL-T_HIGH_ cell-mediated suppression of EAE. Downregulation of *Il10* combined with the upregulation of *Ccr6* could indicate the selective recruitment of IL-10 non-producing CCR6^+^T_H_17 cells among the CD4^+^2D2-IEL-T_HIGH_ cells (Figs 2e and 5a,b). We further confirmed the expression of LAG-3 protein in CD4^+^2D2-IEL-T_HIGH_ cells and CNS-CD4^+^2D2-IEL-T_HIGH_ cells. LAG-3 expression in CD4^+^2D2-IEL-T_HIGH_ cells from the gut was minimal, but its intracellular expression was markedly higher in the CNS-CD4^+^2D2-IEL-T_HIGH_ cells (Fig. 5c), similar to what was previously reported in tumour-infiltrating CD8^+^ T cells^23^. Intracellular expression of Foxp3, minimally detected in CD4^+^2D2-IEL-T_HIGH_ cells (Fig. 2d), was further reduced in CNS-CD4^+^2D2-IEL-T_HIGH_ cells (Fig. 5d), which corresponded with *Foxp3* mRNA expression (Fig. 5a). Collectively, these results indicate that CD4^+^2D2-IEL-T_HIGH_ cells suppress the development of EAE through a mechanism independent of Foxp3-expressing T cells.

### CD4^+^2D2-IEL-T_HIGH_ cells suppress T-cell proliferation

**Figure 6:**
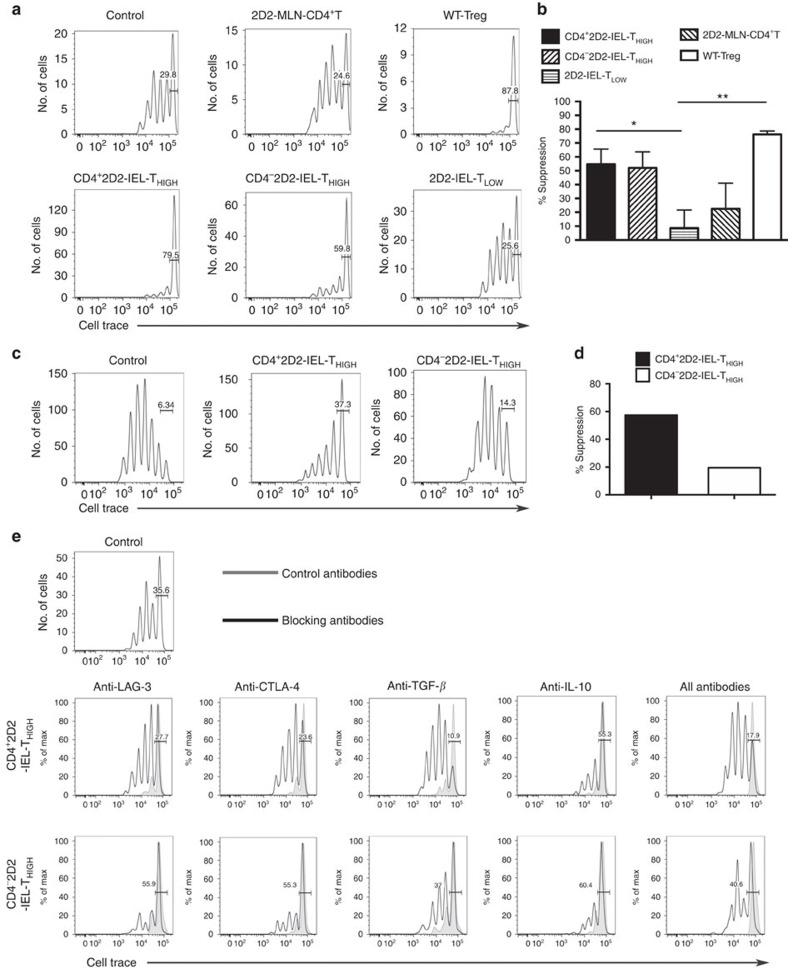
Suppressive effect of CD4^+^2D2-IEL-T_HIGH_ cells on T-cell proliferation *in vitro*. (**a**) Sorted spleen CD4^+^CD25^−^ T cells from 2D2 mice were labelled with CellTrace and were used as responder cells. The indicated suppressor cells were sorted and co-cultured. Suppressor cells were not added to control cultures. CD4^+^Vα3.2^+^Vβ11^+^ cells from MLN of 2D2 mice (2D2-MLN), CD4^+^CD25^+^ T cells from WT spleen cells (Treg) and CD4^+^ or CD4^−^ 2D2-IEL-T_HIGH_ and T_LOW_ cells sorted from IEL populations of 2D2 mice were used as suppressor cells. Cells were stimulated with APCs (CD3^−^ WT spleen cells) plus anti-CD3 mAb. (**b**) Per cent suppression was calculated from three to four samples for each condition from three experiments including **a**. Mean and s.e.m.; *P<0.05, **P<0.01 by unpaired *t*-test. (**c**) The responder cells used in **a** were co-cultured with the indicated suppressor cells and MOG(35-55)-pulsed APCs and average per cent suppression was calculated in **d**. (**e**) Blocking mAb or isotype-matched control antibodies were added to the cell cultures. After 4 days of culture in 96-well U-bottom (**a**–**d**) or V-bottom (**e**) plates, intensities of CellTrace in 7AAD^−^CD4^+^ cells were measured by FACS. Bars represent undivided CellTrace-labelled responder cells. (**c**,**e**) Data are representative of two experiments. FACS, fluorescence-activated cell sorting.

To elucidate the mechanisms of EAE suppression, we tested whether the 2D2-IEL populations could suppress splenic CD4^+^2D2-T-cell proliferation following stimulation with anti-CD3 mAb in the presence of spleen accessory cells *in vitro*. CD4^+^CD25^+^ regulatory T cells from WT mice suppressed responder T-cell proliferation as expected (Fig. 6a,b). CD4^+^ and CD4^−^2D2-IEL-T_HIGH_ cells also suppressed the proliferation of responder CD4^+^2D2-T cells. In contrast, 2D2-IEL-T_LOW_ cells or CD4^+^2D2-T cells from mesenteric lymph nodes (MLN) did not inhibit the proliferation of splenic CD4^+^2D2-T cells. Notably, a substantial proportion of CD4^+^ and CD4^−^2D2-IEL-T_HIGH_ cells survived after co-culture with responder T cells compared with 2D2-IEL-T_LOW_ cells, implicating their proliferation during the assays. The proportion of CD4^+^2D2-IEL-T_HIGH_ cells in cultured T cells was lower and the proportion of CD4^−^2D2-IEL-T_HIGH_ cell was comparable to that of 2D2-MLN-CD4^+^ T cells with no suppressive capabilities (Supplementary Fig. 6 and Fig. 6a,b), indicating that they do not suppress responder T-cell proliferation by over-proliferating.

Next, we used MOG(35-55)-pulsed spleen antigen-presenting cells (APCs) to stimulate cells in the assay. CD4^+^2D2-IEL-T_HIGH_ cells suppressed responder T-cell proliferation, further indicating their ability to suppress T-cell responses. However, CD4^−^2D2-IEL-T_HIGH_ cells showed only a mild effect in this modified assay (Fig. 6c,d). These results are consistent with the findings that adoptive transfer of CD4^+^, but not CD4^−^, 2D2-IEL-T_HIGH_ cells ameliorated MOG(35-55)-induced EAE (Fig. 3f).

To clarify the molecular mechanisms of CD4^+^2D2-IEL-T_HIGH_ cell suppression, we tested the effects of blocking antibodies to CTLA-4, LAG-3, TGF-β and IL-10 on *in vitro* T-cell suppression by IEL cells. Blocking CTLA-4, LAG-3 or TGF-β abrogated the suppressive activity of CD4^+^2D2-IEL-T_HIGH_ cells, whereas blocking IL-10 did not (Fig. 6e). Suppression by CD4^−^2D2-IEL-T_HIGH_ cells was only slightly reversed by blocking TGF-β, and was not reversed by blocking other molecules (Fig. 6e).

### LAG-3 is crucial for EAE suppression by 2D2-IEL-T_HIGH_ cells

**Figure 7:**
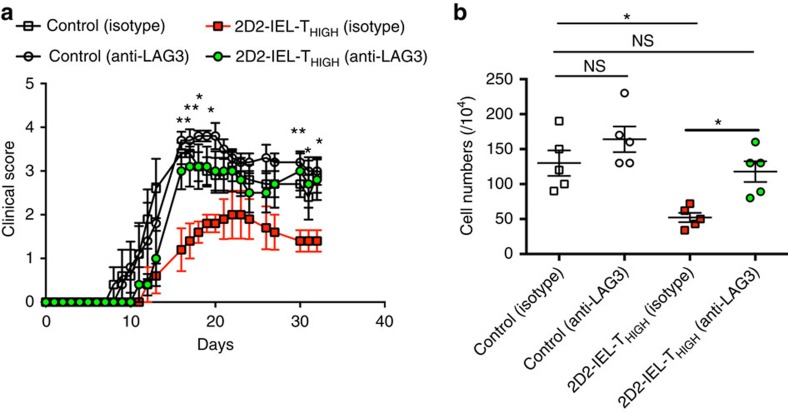
The role of LAG-3 in 2D2-IEL-T_HIGH_ cell-mediated suppression of EAE. (**a**) In total, 1.0 × 10^6^ WT spleen cells (control) or 2D2-IEL-T_HIGH_ cells (2D2-IEL-T_HIGH_) were transferred to WT recipients (*n*=5 per group) one day before EAE induction (day 0). Anti-LAG-3 mAb or isotype-matched immunoglobulin was administrated. Clinical scores of EAE were evaluated (mean and s.e.m). *P<0.05, **P<0.01 by two-way analysis of variance (ANOVA) with Bonferroni's post-test. (**b**) Mononuclear cells infiltrating the CNS of recipient mice were recovered on day 33, and cell numbers were counted. Individual plots represent cell numbers from each animal (mean and s.e.m.). *P<0.05 or NS (not significant) by one-way ANOVA with Bonferroni's post-test. Data are representative of two experiments.

Subsequently, we focused on the involvement of LAG-3 in the suppression of EAE by 2D2-IEL-T_HIGH_ cells, since *Lag3* was the most highly upregulated immunoregulatory gene among the 2D2-IEL-T_HIGH_ cells infiltrating the CNS. We administered LAG-3-blocking mAb or isotype-matched control antibodies into mice with or without transfer of 2D2-IEL-T_HIGH_ cells. Injection of LAG-3-blocking antibody had no significant effect on the severity of EAE clinical disease observed in the control mice (Fig. 7a). However, LAG-3 blockade significantly increased disease severity of EAE in the 2D2-IEL-T_HIGH_ cell recipient mice when compared with mice that received 2D2-IEL-T_HIGH_ cells and then injected with isotype-matched mAb (Fig. 7a). In parallel with disease severity, the number of mononuclear cells infiltrating the CNS was significantly reduced in 2D2-IEL-T_HIGH_ cell recipient EAE mice compared with non-recipient EAE mice. However, LAG-3 blockade in 2D2-IEL-T_HIGH_ cell-transferred EAE mice increased the number of CNS infiltrating mononuclear cells compared with controls (Fig. 7b). These results indicate the critical involvement of LAG-3 in the 2D2-IEL-T_HIGH_ cell-mediated suppression of EAE.

### Suppressive capacity of CD4^+^ IELs in KBx/N mice and WT mice

**Figure 8:**
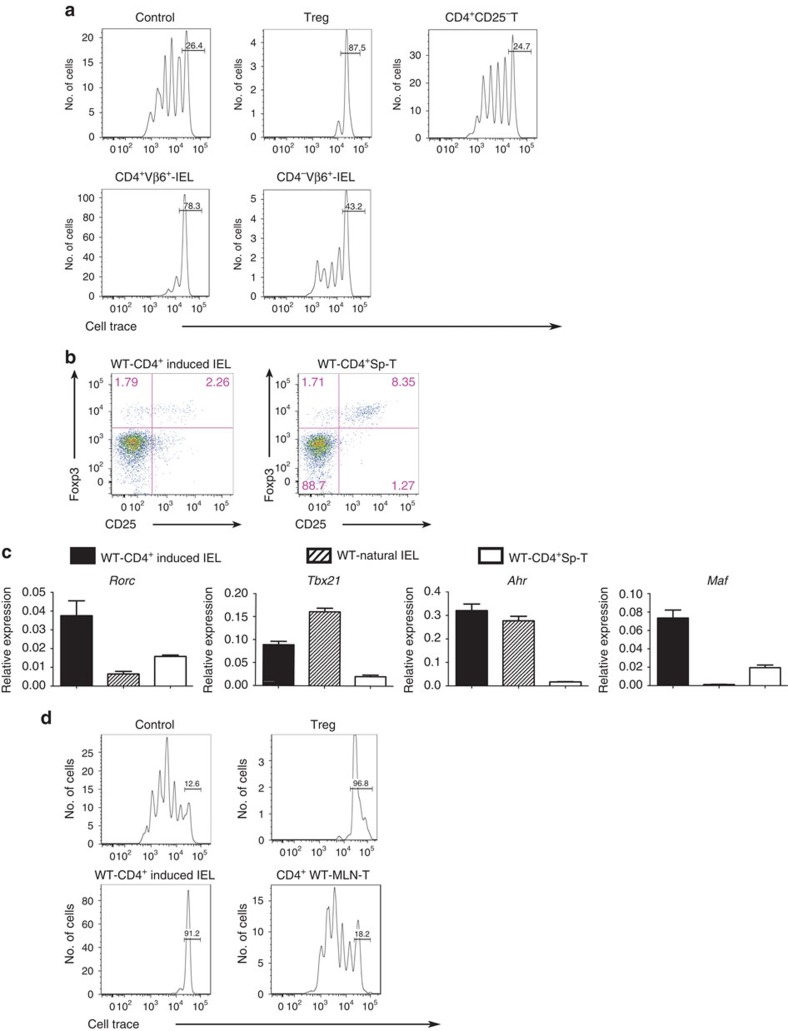
Suppressive capabilities of CD4^+^ IEL from KBx/N mice and WT mice. (**a**) IELs from KBx/N mice were used. Responder CD4^+^CD25^−^ T cells (labelled with CellTrace) and CD3^−^ cells were sorted from the spleens of KBx/N transgene-negative littermates. CD4^+^CD25^−^ T cells sorted from the spleens of transgene-negative littermates were used as responder cells. CD4^+^CD25^+^ T (Treg) cells sorted from the spleen cells of transgene-negative littermates were used as suppressor cells as a positive control. CD4^+^ and CD4^−^ Vβ6^+^ T cells were sorted from KBx/N-IELs and used as suppressor cells. Control data were obtained without adding suppressor cells. Data are representative of three experiments. (**b**) WT-CD4^+^CD8α^−^ induced IEL and WT-CD4^+^Sp-T cells were analysed for the expression of surface CD25 and intracellular Foxp3. Data represent two experiments. (**c**) mRNA expression in WT-CD4^+^ induced IELs (CD2^+^CD5^+^CD4^+^CD8α^−^TCRβ^+^IEL), WT natural IELs (CD2^−^CD5^−^CD4^−^TCRβ^+^IEL), and WT-CD4^+^Sp-T cells was analysed by quantitative RT–PCR (*n*=4) mean and s.e.m. (**d**) Suppressor assay using IELs from WT mice. Responder T cells, CD3^−^ spleen cells, CD4^+^ induced IELs and CD4^+^CD25^+^ T cells (Treg) in the spleen and MLN-CD4^+^ T cells were sorted from WT mice. After 4 days of culture in 96-well V-bottom (**a**) or U-bottom (**d**) plates, intensities of CellTrace in 7-AAD^−^CD4^+^ cells were analysed by FACS. Bars represent undivided CellTrace-labelled responder T cells. Data representative of three experiments are shown. FACS, fluorescence-activated cell sorting; RT–PCR, reverse transcription PCR.

Next, we examined if the regulatory potential of IELs is specifically associated with 2D2 mice or if it is also a characteristic of other mouse strains, such as KBx/N TCR-transgenic mice, that spontaneously develop arthritis^24^. This mouse strain was generated by crossing ribonuclease peptide-reactive KRN TCR-transgenic mice with non-obese diabetic (NOD) mice^24^. We isolated CD4^+^ and CD4^−^ IELs expressing Vβ6^+^ KRN TCR from the small intestines of KBx/N mice and examined their regulatory potential *in vitro*. CD4^+^CD25^+^ regulatory T cells, used as positive controls, suppressed responder T-cell proliferation *in vitro*, whereas CD4^+^CD25^−^ T cells did not (Fig. 8a). CD4^+^Vβ6^+^IEL cells, corresponding to CD4^+^KRN induced IELs, suppressed responder T-cell proliferation similar to CD4^+^CD25^+^ regulatory T cells. However, CD4^−^Vβ6^+^IELs were only partially suppressive, compared with CD4^+^CD25^+^ regulatory T cells.

To investigate the physiological immunoregulation in WT mice, we next compared the phenotype of CD4^+^ induced IELs from WT mice with that of CD4^+^2D2-IEL-T_HIGH_ cells. WT-CD4^+^ induced IELs had a surface phenotype similar to that of CD4^+^2D2-IEL-T_HIGH_ cells (Supplementary Fig. 3a and Fig. 1e,f). Compared with CD4^+^2D2-IEL-T_HIGH_ cells, most of the WT-CD4^+^ induced IELs were CD25^−^; however, there was a small number of CD25^+^Foxp3^+^ regulatory T cells and CD25^+^Foxp3^−^ cells (Fig. 8b). Regarding the expression of T_H_ cell-related transcription factors, WT-CD4^+^ induced IELs resembled CD4^+^2D2-IEL-T_HIGH_ cells (Figs 2c and 8c). We also examined whether WT-CD4^+^ induced IELs could suppress the polyclonal proliferation of WT spleen CD4^+^ T cells *in vitro*. WT-CD4^+^ induced IELs efficiently suppressed responder T-cell proliferation *in vitro*, although CD4^+^ T cells from WT-MLN did not (Fig. 8d). These results indicate that the regulatory phenotype of autoreactive T cells in the gut is not restricted to 2D2 mice, but that this phenotype could be associated with gut-resident autoreactive T cells with alternative antigen specificities and with CD4^+^ induced IELs in WT mice.

### CD4^+^2D2-IEL-T_HIGH_ cells proliferate in the MLN and gut

**Figure 9:**
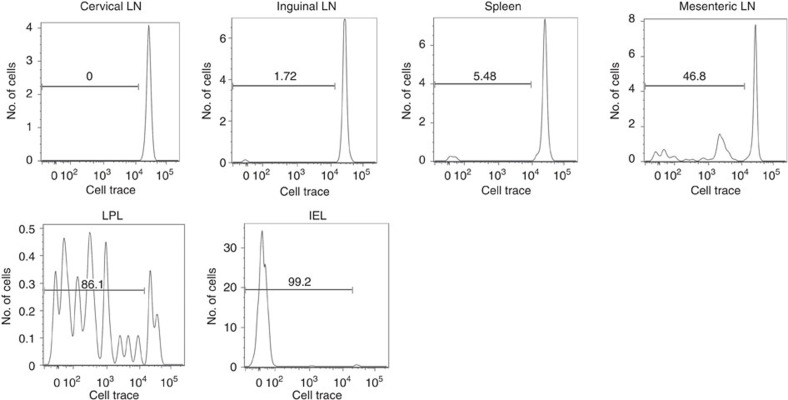
The proliferation of CD4^+^2D2-IEL-T_HIGH_ cells in non-immunized mice. In total, 1.5 × 10^5^ 2D2-IEL-T_HIGH_ cells (CD45.2^+^) were labelled with CellTrace and transferred intravenously to CD45.1^+^WT mice. Five days later, cervical lymph node (LN) cells, inguinal LN cells, MLN cells, spleen cells and small intestinal IELs and LPL were collected. 7AAD^−^CD45.2^+^CD45.1^−^CD4^+^ cells were gated and CellTrace intensities were measured by FACS. Samples are pooled from two mice. Data represent three samples from two experiments. FACS, fluorescence-activated cell sorting.

The existence of CD4^+^2D2-IEL-T_HIGH_ cells in 2D2 mice at steady state and the detection of these cells in recipients 3 weeks after transfer (Fig. 3c,d and Supplementary Fig. 4b) indicate that these cells could have proliferated in non-immunized mice. To address this point, 2D2-IEL-T_HIGH_ cells were labelled with CellTrace Violet dye and transferred to congenic naive WT mice to observe the proliferative capacity of transferred cells in the secondary lymphoid organs, gut and CNS (Fig. 9). CD4^+^2D2-IEL-T_HIGH_ cells detected in the cervical and inguinal lymph nodes or the spleen showed very low proliferation. On the contrary, CD4^+^2D2-IEL-T_HIGH_ cells detected in the MLN exhibited a clear proliferative response. Moreover, transferred CD4^+^2D2-IEL-T_HIGH_ cells detected in the gut lamina propria showed significant proliferation. The transferred cells within the IEL population showed very low CellTrace intensity, indicating that they had vigorously proliferated in the lamina propria before reaching the epithelium. Almost no transferred CD4^+^2D2-IEL-T_HIGH_ cells were detected in the CNS (Supplementary Fig. 7). These results indicate that CD4^+^2D2-IEL-T_HIGH_ cells can proliferate in the gut-associated lymphoid tissues and gut tissue of non-immunized mice via antigens derived from the intestinal contents. This is presumably important for their induced IEL phenotype and robust suppressive effect *in vivo*.

### Gut microbiota and dietary AHR ligands induce CD4^+^ IELs

**Figure 10:**
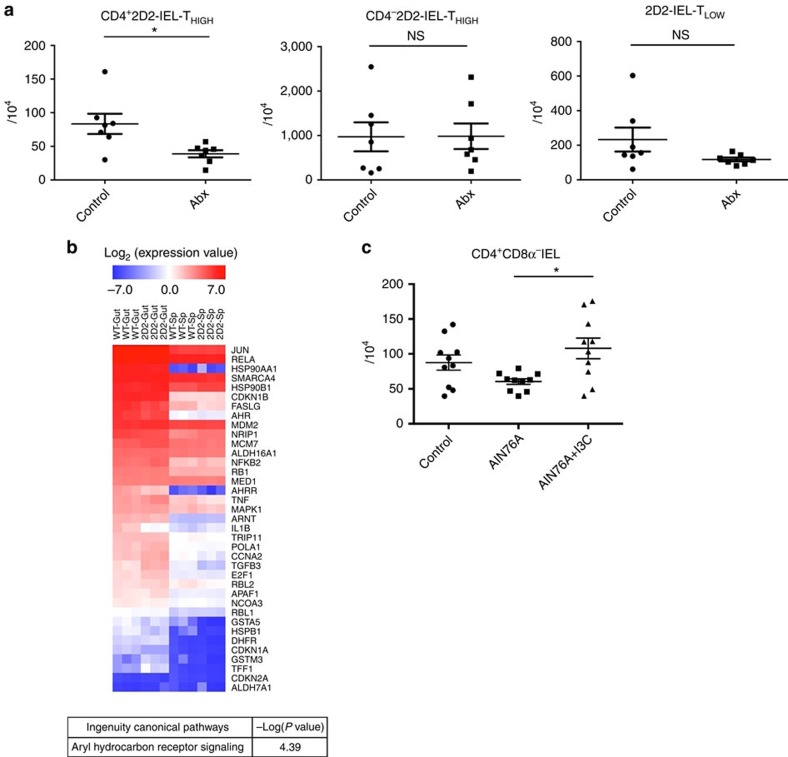
The contribution of gut environmental stimuli to the induction of CD4^+^ IELs. (**a**) Six-week-old 2D2 mice were orally treated with distilled water (control) or a mixture of antibiotics (Abx) (*n*=7 per group). Two weeks later, IELs were collected and analysed for absolute numbers of 2D2-IEL cells. Each dot represents an individual mouse. Data are pooled from two experiments (mean and s.e.m.). (**b**) The microarray gene expression analysis showed a pattern of higher expression in WT-CD4^+^CD8α^−^ CD25^−^IEL (WT-Gut) and CD4^+^CD25^−^2D2-IEL-T_HIGH_ cells (2D2-Gut) compared with WT-CD4^+^CD25^−^ spleen-T cells (WT-Sp) and CD4^+^CD25^−^2D2-spleen-T cells (2D2-Sp). The log_2_ expression values of AHR signalling pathway-related genes are shown in the heatmap. Each column represents a single sample (*n*=3 per group) and each row represents a single gene (38 genes). The result of the ingenuity pathway analysis is shown below (see Method). (**c**) A standard diet (control), synthetic purified diet (AIN76A) or synthetic diet supplemented with I3C (AIN76A+I3C) was fed to WT mice. After 1 month, IELs were obtained and the absolute numbers of CD4^+^CD8α^−^TCRβ^+^ IELs were analysed (*n*=10 per group). Each dot represents an individual animal. Pooled data from two experiments are shown (mean and s.e.m.). (**a**) *P<0.05 by unpaired *t*-test. (**c**) *P<0.05 by one-way analysis of variance with Bonferroni's post-test.

Finally, we investigated the gut environmental stimuli that contribute to the development of regulatory CD4^+^ IELs. First, we investigated whether the generation of CD4^+^2D2-IEL-T_HIGH_ cells is dependent on the gut microbiota. To deplete a broad spectrum of bacterial species, we orally treated 2D2 mice with a mixture of antibiotics. Oral antibiotic treatment significantly reduced the absolute CD4^+^2D2-IEL-T_HIGH_ cell numbers, whereas the numbers of CD4^−^2D2-IEL-T_HIGH_ or 2D2-IEL-T_LOW_ cells were not significantly altered (Fig. 10a). These data indicate that the gut microbiota is involved in the induction of CD4^+^2D2-IEL-T_HIGH_ cells, in accordance with the phenotype of induced IELs^16^,^25^.

AHR signalling has been suggested to modulate a broad array of genes, including factors involved in cell proliferation and survival. Notably, exogenous AHR stimuli were reported to be critical for the maintenance of γδ^+^ IELs^26^. Therefore, we investigated the involvement of dietary AHR ligands in the development of CD4^+^ IELs. Microarray data revealed that the number of AHR signalling pathway-related genes is similarly high in CD4^+^ IELs derived from WT and 2D2 mice when compared with spleen CD4^+^ T cells, indicating that signalling from AHR ligands in the intestine may modulate the development of CD4^+^ IELs (Fig. 10b and Supplementary Fig. 8). Next, we fed WT mice with our standard diet, a synthetic purified diet that includes low levels of AHR ligands, and a synthetic diet supplemented with I3C, which is found in cruciferous vegetables and can be converted into high-affinity AHR ligands through exposure to stomach acid^26^,^27^. A tendency towards reduced numbers of CD4^+^ IEL cells was seen in synthetic diet-fed mice compared with control mice. Furthermore, supplementation of I3C in the synthetic diet significantly increased the numbers of CD4^+^ IEL cells when compared with the synthetic diet without the addition of I3C (Fig. 10c). These data suggest that dietary AHR ligands contribute to the induction of CD4^+^ IELs.

Collectively, these results indicate that CD4^+^ induced IELs, which have a regulatory function in CNS autoimmunity, are influenced by gut environmental stimuli such as the microbiota and dietary AHR ligands.

## Discussion

In the present study, we demonstrated that autoreactive CD4^+^ induced IELs exhibit suppressive activities against a T cell-mediated autoimmune disease, that is, EAE. The regulatory IELs were influenced by stimuli from the gut environment, such as the microbiota and dietary AHR ligands. The IELs appear to be induced by MOG(35-55)-specific T cells in response to non-self-antigens derived from the intestinal contents. Therefore, our study provides a clue to identifying the dietary factors and microbiota responsible for the increased incidences of autoimmune diseases including MS. Furthermore, similar regulatory CD4^+^ IELs are present in WT mice and another TCR-transgenic (KBx/N) mouse strain, indicating that autoreactive CD4^+^ IELs that cross-react with intestinal antigens could be pivotal in the prevention of autoimmune diseases. Supporting this concept, previous studies have demonstrated that exogenous antigens, including microbial and viral peptides^28^,^29^, as well as dietary elements such as milk protein butyrophilin, stimulate myelin-reactive T cells^30^. Moreover, it was recently reported that retina-specific T cells are activated by commensal bacteria^31^. The presence of abundant induced IEL cells in TCR-transgenic mice indicates that the pathogenic or regulatory autoimmune T-cell repertoire may actually be shaped by encounters with intestinal antigens.

We show that a dietary AHR ligand, I3C, increases CD4^+^ IELs. I3C is produced in cruciferous vegetables such as cabbage, cauliflower and Brussels sprouts^27^. It is of interest that I3C increased CD4^+^ IELs in an antigen-independent manner, which indicates the possibility of a common dietary element that influences the induction of regulatory IELs in individuals with a variety of human leukocyte antigen alleles. In addition, the function of AHR in T cells depends on the specific ligands bound to the receptor^32^,^33^. Thus, the effect of I3C or other AHR ligands on CD4^+^ IEL function may be a worthwhile avenue for further investigation.

Most of the 2D2-CD4^+^ IEL cells were Foxp3 negative, and as few as 2,500–7,000 Foxp3^+^ cells were included in the transfer experiments. The cell number capable of suppressing EAE reported for antigen-specific regulatory T cells was no less than 1.0 × 10^5^, far from the cell numbers included in our experiments^34^. Moreover, the majority of Foxp3^+^ cells in the CD4^+^2D2-IEL-T_HIGH_ population were CD25 negative. It was recently reported that CD25 negative regulatory T cells lose Foxp3 and convert into pathogenic T_H_17 cells called ‘exFoxp3T_H_17 cells' after transfer into an autoimmune arthritis model^20^. We assume that most of the Foxp3^+^ cells in the CD4^+^2D2-IEL-T_HIGH_ population converted into exFoxp3T_H_17 cells lacking regulatory functions.

Moreover, the IL-17-producing CD4^+^ induced IELs differ from Foxp3^+^ regulatory T cells and are distinct from regulatory T_H_17 cells that co-produce IL-17 and IL-10 (refs 14, 21). IL-10 production is regarded as a major suppressive mechanism for regulatory T_H_17 cells and T regulatory type 1 (Tr1) cells co-expressing CD49b and LAG-3 (refs 35, 36). However, 2D2-CD4^+^ IELs are unique in that they do not use IL-10 as a critical mediator for suppression and critically depend on LAG-3, which is upregulated in the CNS.

LAG-3 plays a downregulatory role in autoimmune diseases models of myocarditis in PD-1-deficient BALB/c mice and type 1 diabetes mellitus in NOD mice^37^,^38^. LAG-3 is expressed by Foxp3^+^ regulatory T cells and required for optimal regulatory T-cell function^39^. Of note, major histocompatibility complex class II engagement by LAG-3-expressing regulatory T cells suppressed the activation of dendritic cells^40^. We speculate that there is a similar role for LAG-3 in the suppression by CD4^+^ induced IELs. We also demonstrated that CTLA-4 and TGF-β are involved in the suppression by CD4^+^2D2-IEL-T_HIGH_ cells. CTLA-4 is a cell-intrinsic and -extrinsic regulator of T-cell responses, and its expression in activated T cells and Foxp3^+^ regulatory T cells is known to restrict autoimmune pathology^41^.

ThPOK suppresses Runx3, which induces cytotoxic T-lymphocyte programmes in CD4^+^ T cells^42^ and promotes production of IFNγ from T_H_1 cells^43^. The loss of ThPOK and the expression of Runx3 by gut CD4^+^ IELs were previously shown to be antigen-driven processes^42^. However, although cross-reactive antigens were assumed to exist in the gut and cognate antigens do exist in the CNS, CD4^+^2D2-IEL-T_HIGH_ cells had reduced but sustained expression of ThPOK and did not express Runx3, either in the gut or in the CNS. Regulatory CD4^+^ IELs may have an intrinsic mechanism to maintain the expression of ThPOK, preventing them from acquiring cytolytic function and producing excess IFNγ.

The mechanism by which CD4^+^2D2-IEL-T_HIGH_ cells acquire their regulatory ability remains to be determined. However, we speculate that they might have gained the potential for immunoregulation during their encounter with intestinal cross-reactive antigens in the MLNs and the intestine. In support of this, Cassani *et al*. previously demonstrated by using mice deficient for CCR9, β7 or blocking antibodies against MAdCAM-1 that migration of CCR9^+^α4β7^+^ T cells to the intestinal mucosa is critically involved in oral tolerance^44^. They proposed that regulatory T cells stimulated in the MLNs migrate to the gut mucosa, wherein they undergo a second activation step to differentiate completely into IL-10-producing regulatory T cells. Following the second stimulation in the gut, the regulatory T cells return to systemic circulation to counter proinflammatory responses. Determining whether this model is applicable to the full maturation of autoimmune regulatory CD4^+^ IELs is of interest.

Gut-resident CD4^+^2D2-IEL-T_HIGH_ cells express various activation markers, but not CD25, as well as co-stimulatory molecules associated with activated T cells. Such ‘partially activated' cells can become fully activated immediately on appropriate TCR and co-stimulatory signalling^45^. The unique phenotype of the CD4^+^2D2-IEL-T_HIGH_ cells in the gut could be maintained by co-stimulatory and cytokine signals, such as TGF-β signalling, provided by gut epithelial cells and mononuclear phagocytes^46^,^47^,^48^.

We demonstrated that the CD4^+^ induced IEL would differentially express key suppressive molecules in the gut epithelium and inflammatory CNS tissue. In fact, LAG-3 was highly upregulated after the cells entered the CNS, whereas CTLA-4 and TGF-β were constitutively expressed. It is possible that before LAG-3 is fully upregulated in the CNS, regulation through CTLA-4 is critical. Since the peripheral effect of the transfer of 2D2-IEL-T_HIGH_ cells was slight, we assume that the change to an enhanced regulatory phenotype mainly occurs through signals from the inflammatory CNS foci, especially from the CNS-APCs that present cognate antigens and express high levels of co-stimulatory molecules^49^.

The upregulation of LAG-3 in regulatory CD4^+^ IELs was limited to the intracellular portion. It was previously reported that the expression of LAG-3 in tumour-infiltrating CD8^+^ T cells was also limited to intracellular stores. Nevertheless, blockade by anti-LAG-3 antibody had a robust effect^23^. This is a situation similar to that of CTLA-4, in which the surface portion on activated T cells is rapidly endocytosed and higher levels of expression are detected in the intracellular portion^50^. The same molecular mechanism is assumed to underlie the distribution of LAG-3 in CD4^+^ T cells as in CD8^+^ T cells and CTLA-4.

A previous study of parabiotic mice indicated that although CD8^+^ IELs will not recirculate, CD4^+^IELs and lamina propria lymphocytes (LPL) might recirculate in the blood^51^. Recent studies using photoconversion mice showed that both T and B cells in the gut could enter the bloodstream and subsequently traffic to secondary lymphoid organs^52^,^53^, suggesting that regulatory CD4^+^ IEL cells could also recirculate with other T cells, reach the inflammatory tissues and upregulate LAG-3, thereby preventing the acceleration of pathological autoimmune inflammation. In this regard, it is of note that CD4^+^2D2-IEL-T_HIGH_ cells express *Ccr6*, *Ccr2* and *Cxcr3*, which promote T-cell migration into the inflamed CNS^54^,^55^,^56^. We also demonstrated that CD4^+^ induced IELs with TCRs capable of causing arthritis and those from WT mice had a phenotype and suppressive capability similar to those associated with CD4^+^2D2-IEL-T_HIGH_ cells. Taken together, it is reasonable to postulate that CD4^+^ T cells with autoimmune TCRs in the gut epithelia have a physiological role in the regulation of autoimmune diseases.

In summary, our results indicate a new role for CD4^+^ IELs in regulating CNS inflammation. The IELs comprise a significant proportion of the peripheral T-cell pool^16^ and are subject to environmental stimuli such as the gut microbiota and dietary elements. Dysbiosis or insufficient AHR ligands in the diet may reduce the induction of such regulatory cells, thus promoting the development of autoimmune diseases such as MS. Further studies of human regulatory IELs hold promise for possible clinical applications.

## Methods

### Mice

C57BL/6 mice were purchased from CLEA Laboratory Animal Corp. 2D2 TCR-transgenic mice were purchased from Jackson Laboratories. KRN TCR-transgenic mice were kindly provided by Diane Mathis and Christophe Benoist (Harvard Medical School Boston, MA). KRN mice were crossed with NOD mice to generate KBx/N mice. Six- to ten-week-old mice were used. Mice were maintained in specific pathogen-free conditions in accordance with the institutional guidelines. This study was approved by the Committee for Small Animal Research and Animal Welfare (National Center of Neurology and Psychiatry).

### Cell preparation

Small intestinal IELs were isolated as previously described with some modifications^57^. Small intestines were removed and Peyer's patches were dissected carefully. They were opened longitudinally, washed vigorously and then cut into 0.5-cm pieces. Tissues were washed and incubated in Ca^2+^- and Mg^2+^-free Hank's buffered saline (HBSS) containing 15 mM HEPES and 5% FBS (all from Gibco) in the presence of 0.5 mM EDTA and 2 mM dithiothreitol (GE Healthcare) at 37 °C for 40 min with shaking. The supernatants from released cells were passed through 70- and 40-μm cell strainers, suspended in 44% Percoll (GE Healthcare), overlaid onto 67% Percoll and centrifuged for 20 min at 970*g* at 4 °C. Cells at the interface were collected as IEL. To obtain LPL, the remaining tissues were further washed and incubated in HBSS containing 5 mM EDTA, 15 mM HEPES and 5% FBS for 30 min with shaking. Subsequently, the tissues were digested in RPMI 1640 medium (Gibco) containing 15 mM HEPES, 1.0 mg ml^−1^ Collagenase D (Roche) and 15 μg ml^−1^ DNase I (Roche) for 50 min with shaking. The resulting tissue homogenates were passed through 70- and 40-μm cell strainers, suspended in 40% Percoll, loaded onto 80% Percoll and centrifuged for 20 min at 970*g* at 4 °C. Cells at the interface were used as LPL.

CNS-infiltrating mononuclear cells were isolated from spinal cords and brains as follows. CNS tissues were cut into small pieces and digested in RPMI 1640 medium (Gibco) containing 1 mM HEPES, 1.2 mg ml^−1^ Collagenase H (Roche) and 100 μg ml^−1^ DNase I (Roche) at 37 °C for 45 min with rotation. The resulting tissue homogenates were passed through a 70-μm strainer and suspended in 40% Percoll, overlaid on 80% Percoll and centrifuged for 20 min at 970*g* at 4 °C. Cells at the interface were CNS mononuclear cells.

Single-cell suspensions from lymph nodes and spleen were obtained by mechanical disruption. For spleen cells, ACK lysis buffer was used to remove red blood cells.

### Adoptive transfer

The indicated numbers of sorted IEL cells from 2D2 mice or spleen cells obtained from WT (C57BL6) mice were injected with PBS intraperitoneally or intravenously into WT recipients on the day before EAE induction.

### Surface or intracellular staining and cell sorting

Nonspecific staining was inhibited by incubation with anti-CD16/32 (BioLegend). Cells were then stained with fluorescence-labelled antibodies. Antibodies against CD45 (30-F11), TCRβ (H57-597), Vα3.2 (RR3-16), Vβ11 (KT11), Vβ6 (RR4-7), CD4 (RM4-5, GK1.5), CD8α (53-6.7), CD8β.2 (53-5.8), CD2 (RM2-5), CD5 (53-7.3), CD25 (3C7), CD3ɛ(145-2C11) and LAG-3 (C9B7W) were purchased from BioLegend. Antibodies against CD69 (H1.2F3), CD103 (M290), CD62L (MEL-14), CD44 (IM7) and CD25 (PC61) were purchased from Becton Dickinson. Dead cells were stained by 7-AAD viability staining solution (BioLegend). For intracellular cytokine staining, cells were stimulated with phorbol 12-myristate 13-acetate and ionomycin at the indicated concentrations (Life Technologies) with GolgiStop for 5 h before staining with anti-IL-17A, IL-10 and IFNγ (Becton Dickinson) using a Cytofix/Cytoperm Kit (Becton Dickinson). Intracellular FoxP3 was stained using an APC Anti-Mouse/Rat FoxP3 staining set (eBioscience) according to the manufacturer's protocol. Intracellular LAG-3 or Ki67 was stained using a Cytofix/Cytoperm Kit followed by staining with LAG-3/Ki67 (ref. 23). Cells were analysed or sorted by BD FACS Canto II or FACS Aria II. All the antibodies were used at a dilution of 1:100.

### Proliferation and cytokine analysis

For proliferation and cytokine measurements, cells were suspended in RPMI 1640 medium supplemented with 10% FBS, 2 mM L-glutamine, 100 U ml^−1^ of penicillin–streptomycin and 50 μM 2-mercaptoethanol (2-ME) (Gibco) and stimulated with immobilized anti-CD3 (2C11, 3.0 μg ml^−1^) and anti-CD28 (37.51, 1.0 μg ml^−1^; Becton Dickinson) for 3 days in 96-well flat-bottom plates. Cells were incubated with [^3^H] thymidine (1 μCi per well) for the final 8 h of culture and incorporation of radioactivity was analysed by a scintillation counter and expressed as counts per minute. Supernatants were collected and cytokines were measured using the Cytometric Bead Array Flex Set (Becton Dickinson). Assays were conducted in triplicate wells.

### EAE induction

For EAE induction, mice were injected subcutaneously with 100μg MOG(35–55) peptide (Toray Research Center, Tokyo, Japan) and 1mg heat-killed Mycobacterium tuberculosis H37RA emulsified in complete Freund's adjuvant (Difco, KS, USA). Two hundred nanograms of pertussis toxin (List Biological Laboratories, CA, USA) was injected intraperitoneally on days 0 and 2 after immunization. The clinical symptoms of EAE were scored (0, no clinical signs; 1, tail weakness; 2, flaccid tail; 3, partial hind limb weakness; 4, total hind limb paralysis; 5, hind and fore leg paralysis).

### Recall responses

For *in vitro* re-stimulation experiments, mice were immunized with MOG(35-55) peptide in complete Freund's adjuvant without pertussis toxin. In total, 3 × 10^5^ cells were collected from dLN and were stimulated with either MOG(35-55) (1–100 μg ml^−1^ for proliferation studies and 100 μg ml^−1^ for cytokine analysis) or with soluble anti-CD3 mAb (1.0 μg ml^−1^) for 3 days in RPMI medium in 96-well U-bottom plates. Cell proliferation and cytokines in the supernatants were measured as described.

### Histological and immunohistological analyses

Mice were perfused and fixed with 4% paraformaldehyde, and the small intestines or spinal cords were obtained. For haematoxylin and eosin and Luxol fast blue staining, tissue specimens were embedded in paraffin and stained according to standard histological procedures. For immunohistochemistry, tissues specimens were embedded in OCT compound (Sakura), flash-frozen and cut on a cryostat (Leica) to yield 12-μm frozen sections. Slides were hydrated in PBS and blocked with 10% goat serum (Jackson ImmunoResearch), incubated with rat anti-Vβ11-biotin (KT11, Abcam) primary antibody at a dilution of 1:100 and detected with Alexa Fluor 488 streptavidin (Life Technologies) at a dilution of 1:400. Slides were mounted with Vectashield Hard Set Mounting Medium with 4,6-diamidino-2-phenylindole (Vector Laboratories). Images were collected by confocal fluorescence microscopy FV1000 (Olympus) at room temperature.

### Quantitative RT–PCR analysis

Sorted cell populations were lysed in RLT buffer (Qiagen) containing 1% 2-ME and subsequently homogenized using a QIA shredder (Qiagen). Total RNA was extracted with RNeasy Mini or Micro kits (Qiagen). Complementary DNA was generated using a SuperScript VILO cDNA Synthesis Kit (Invitrogen) and was used as a template for quantitative RT–PCR performed with the LightCycler FastStart DNA Master SYBR Green I kit (Roche). Primers for *Lag3*, *Ahr*, *Gata3* and *Tbx21* were obtained from Qiagen. Other primers were designed as shown in Supplementary Table 1.

### *In vivo* treatment with anti-LAG-3 antibody

For *in vivo* neutralization of LAG-3, 200 μg of anti-LAG-3 mAb (C9B7W, BioLegend) or purified rat IgG (Invitrogen) in PBS were injected intraperitoneally on day −1 and +1 of EAE induction.

### Suppression assays

FACS-sorted CD4^+^CD25^−^TCRβ^+^ cells from WT- or 2D2-spleen cells were labelled using a CellTrace Violet Cell Proliferation Kit (Invitrogen) following the manufacturer's protocol and were then used as responder T cells. Sorted CD3^−^ spleen cells were irradiated (35 Gy) and used as APCs or pulsed with 50 μg ml^−1^ of MOG(35-55) for 16 h and then irradiated (35 Gy) to use as MOG(35-55)-pulsed APCs. Responder T cells (2 × 10^4^ ) were cultured with 1 × 10^5^ APCs and 2.0 × 10^4^ of the indicated suppressor cells in 96-well plates. Cells were either stimulated with anti-CD3 mAb (1 μg ml^−1^) or with MOG(35-55)-pulsed APCs. For neutralizing experiments *in vitro*, anti-TGF-β (1D11), anti-CTLA-4 (9H10, R&D), anti-IL-10 (JES5-2A5, Abcam) or anti-LAG-3 (C9B7W, Becton Dickinson) antibodies were added to cell cultures at a concentration of 5 μg ml^−1^. Identical concentrations of isotype-matched rat IgG2a, rat IgG1κ or mouse IgG1κ (Becton Dickinson) were used as controls. After 4 days of culture in RPMI medium, cells were collected, stained and the CellTrace intensity of 7-AAD^−^CD4^+^ cells was analysed by FACS. Percentages of proliferated cells (% proliferation) were analysed by FlowJo software. Per cent suppression was calculated as 100–(% proliferation with suppressors/% proliferation of control) × 100 as previously described^58^.

### Oral antibiotic treatment

Mice were orally treated with a mixture of kanamycin sulphate (10 mg), colistin sulphate (2.6 mg) and vancomycin hydrochloride (3 mg) dissolved in 200 μl of distilled water every day using a gavage needle.

### Diets

Mice were fed with a standard diet (CLEA Rodent Diet CE-2; CLEA Japan), a synthetic purified diet (AIN76A Rodent Diet; Research Diets) or a synthetic diet supplemented with 200 p.p.m. of I3C (Sigma-Aldrich) for 1 month.

### Microarray analysis

For the gene expression analysis, samples were sorted in QIAzol Lysis Reagent (QIAGEN), and total RNA was obtained using an miRNeasy Micro kit. Agilent SurePrint G3 Human Gene Expression microarray chips were used according to Agilent protocols. Data were analysed by GeneSpringGX 12.0 (Agilent). After normalization of the expression data, *K*-means clustering was performed to select the genes with the expression pattern as indicated. Among the selected genes, AHR signalling-related genes were further investigated using IPA software and –log (*P* value) was calculated. A heatmap was created with MeV software.

### Statistical analysis

Differences between data groups were analysed by one-way or two-way analysis of variance and Student's *t*-test as indicated. *P* values of <0.05 were considered significant.

## Additional information

**Accession codes**: The microarray data has been deposited in the National Center for Biotechnology Information Gene Expression Omnibus (http://www.ncbi.nlm.nih.gov/geo) under accession number GSE79748.

**How to cite this article:** Kadowaki, A. *et al*. Gut environment-induced intraepithelial autoreactive CD4^+^ T cells suppress central nervous system autoimmunity via LAG-3. *Nat. Commun.* 7:11639 doi: 10.1038/ncomms11639 (2016).

## Supplementary Material

Supplementary Information Supplementary Figures 1-8 and Supplementary Table 1.
