# Supplementary material for: Gut environment-induced intraepithelial autoreactive CD4+ T cells suppress central nervous system autoimmunity via LAG-3
Source: Nat Commun. 2016 May 20;7:11639. doi: 10.1038/ncomms11639 (PMC4876462; doi:10.1038/ncomms11639)
Supplement: Supplementary Information — Supplementary Figures 1-8 and Supplementary Table 1. [file ncomms11639-s1.pdf]

Supplementary Materials

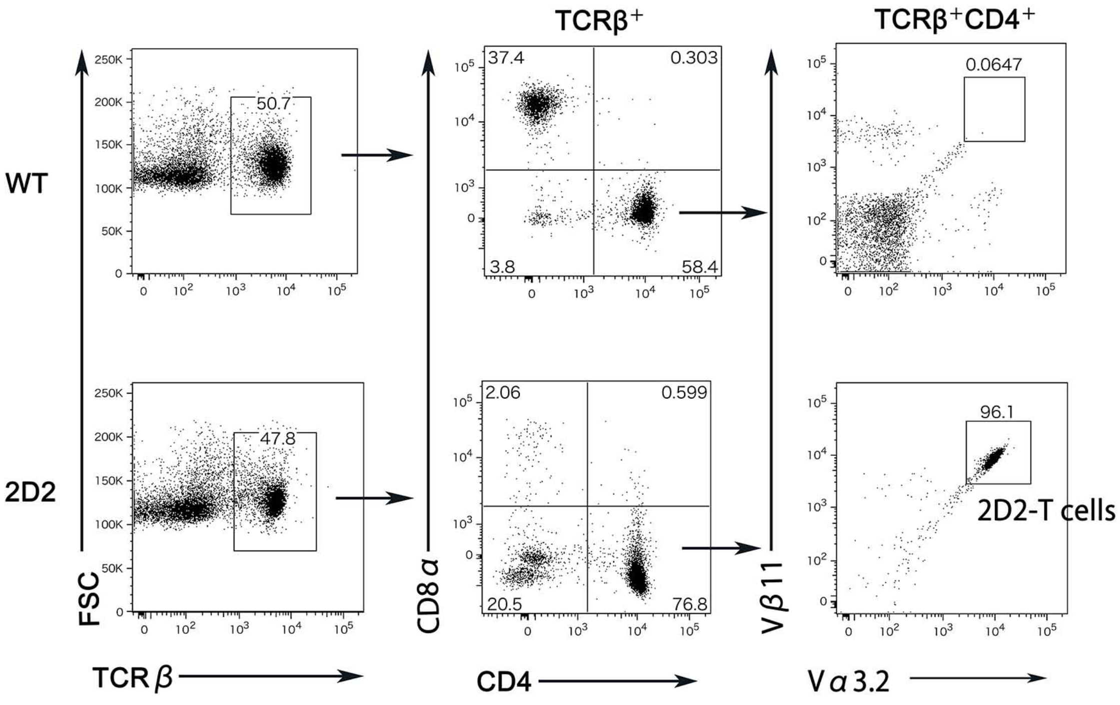

Supplementary Fig. 1: Gating strategy for Vα3.2^{+}Vβ11^{+} 2D2-T cells in spleen cells.

Proportions of Vα3.2^{+}Vβ11^{+} cells among CD4^{+}TCRβ^{+} spleen cells were evaluated by FACS in WT (upper row) and 2D2 (lower row) mice. Representative data from more than three experiments are shown.

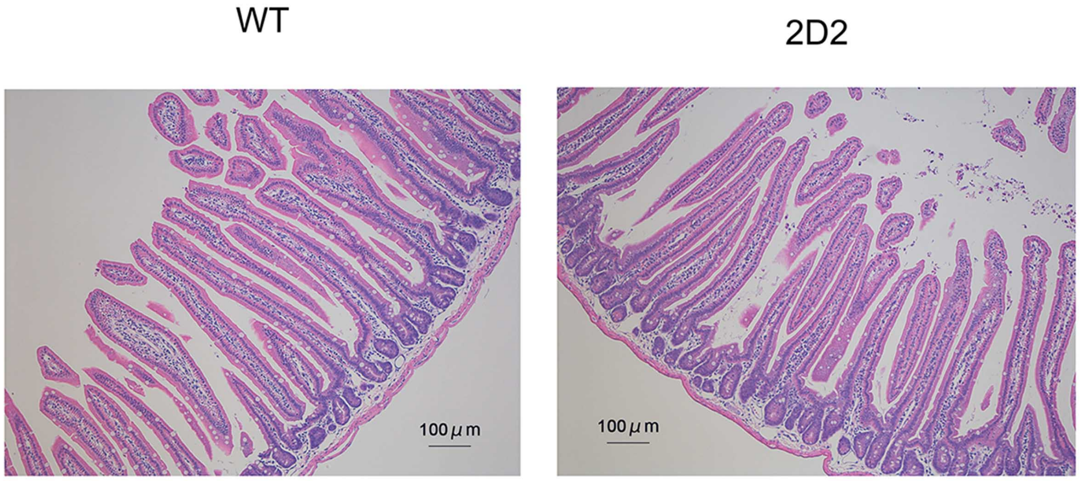

Supplementary Fig. 2: Histology of the small intestines from 2D2 and WT mice.
Histology of the small intestines from WT and 2D2 mice. Paraffin sections were stained with hematoxylin and eosin (H&E). Original magnification: 100×.

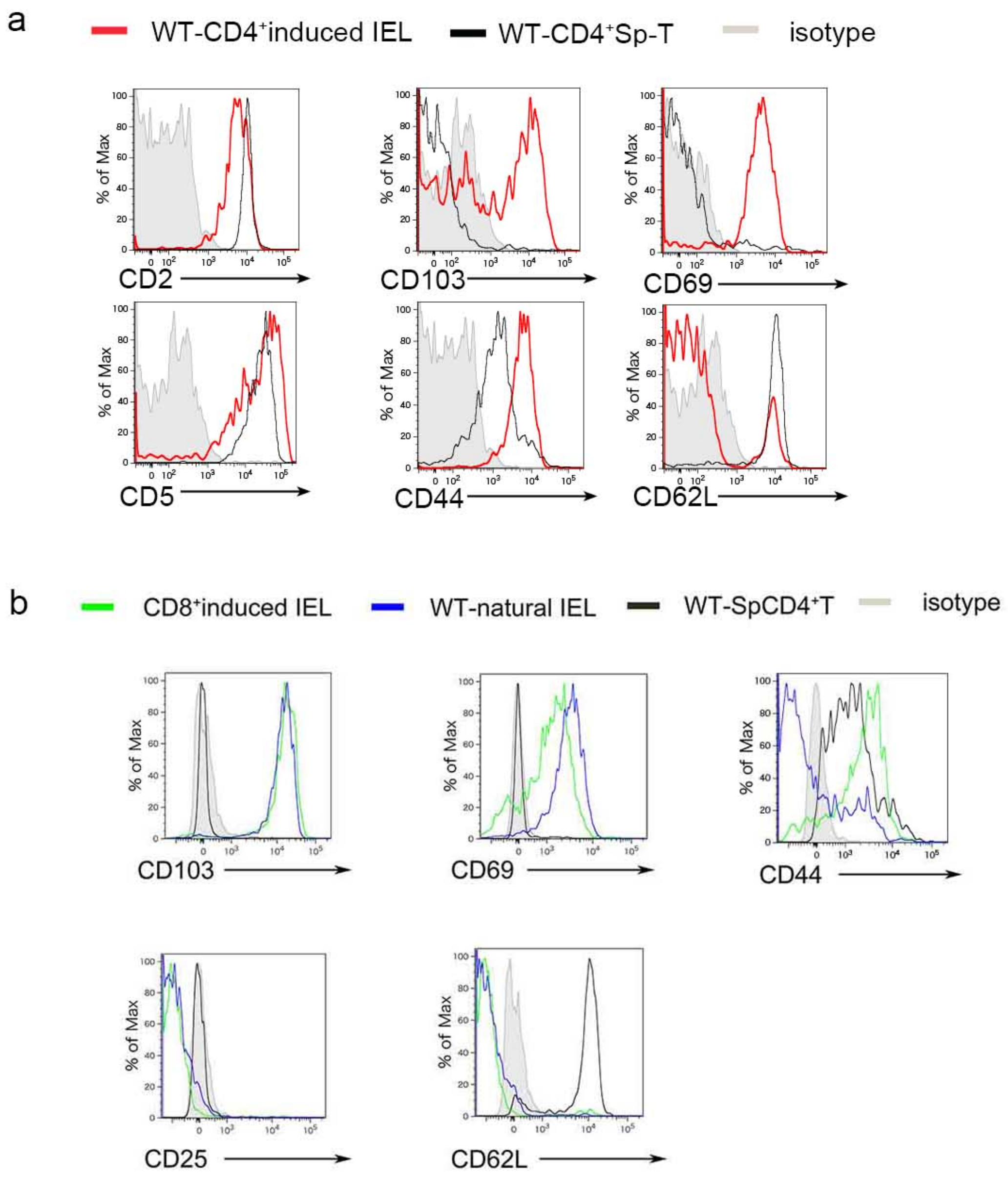

Supplementary Fig. 3: Characterization of WT-IELs.

(**a**) IELs were isolated from WT mice, and the expression of CD2, CD5, CD103, and T cell activation markers was analysed by FACS for WT-CD4$^+$CD8α$^-$TCRβ$^+$ IEL cells (WT-CD4$^+$ ‘induced’ IELs) in comparison with WT-spleen CD4$^+$ TCRβ$^+$ cells (WT-CD4$^+$Sp-T).

(**b**) IELs were isolated from WT mice, and the expression of CD103 and T cell activation markers was analysed by FACS for WT-CD4$^-$CD8β$^+$TCRβ$^+$ IEL cells (WT-CD8$^+$ ‘induced’ IELs) and WT-CD4$^-$CD8β$^-$TCRβ$^+$IEL(WT- ‘natural’ IELs) in comparison with WT-spleen CD4$^+$ TCRβ$^+$ cells (WT-CD4$^+$Sp-T).

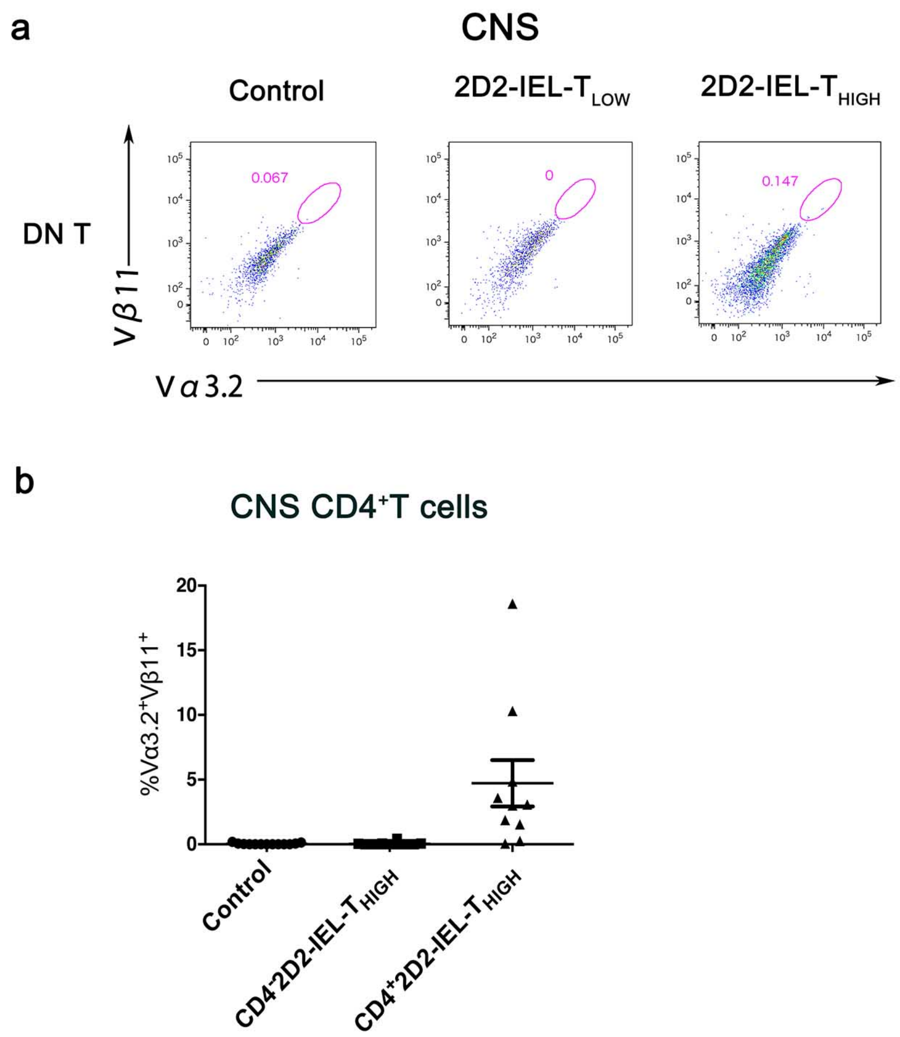

Supplementary Fig. 4: 2D2-TCR$^+$ cells among CNS-T cells of 2D2-IEL-transferred EAE mice. (**a**) Representative results from a flow cytometry analysis of 2D2-TCR (Vα3.2$^+$Vβ11$^+$) expression in DN T cells (CD4$^-$CD8α$^-$TCRβ$^{+)}$ obtained from the CNS of EAE mice (in Fig. 3d) after peak disease. (**b**) CNS mononuclear cells were recovered from the recipients of CD4$^-$ or CD4$^+$ 2D2-IEL-T$_{HIGH}$ cells transferred on day 33 (in Fig. 3e). The proportion (%) of Vα3.2$^+$Vβ11$^+$ cells among CD4$^+$ T cells was analysed by FACS. Each plot shows data from an individual animal (mean, s.e.m.).

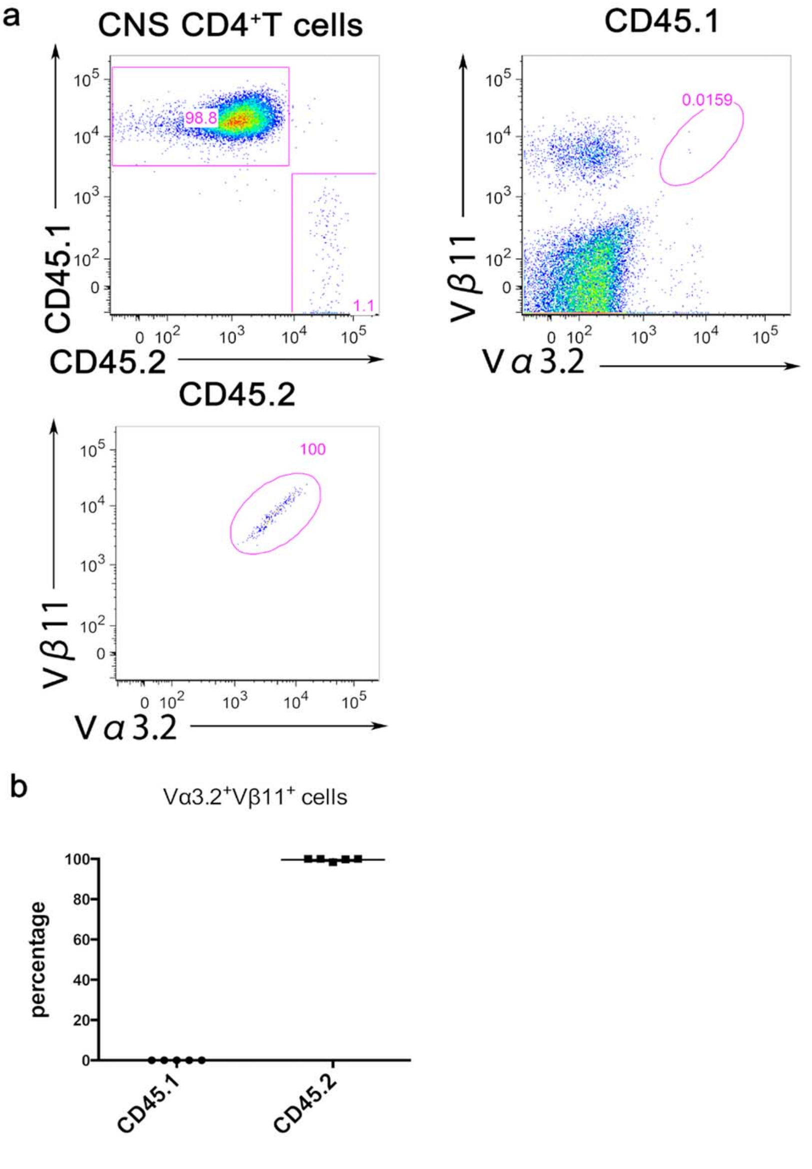

Supplementary Fig. 5: Vα3.2$^+$Vβ11$^+$ cells infiltrating the inflamed CNS of 2D2-IEL-T$_{HIGH}$ cell recipient mice unequivocally represent the transferred cells.

(a) In total, 5.0×10^5 2D2-IEL-T$_{HIGH}$ cells (CD45.2) were transferred intraperitoneally to congenic CD45.1$^+$WT mice (day -1), and EAE was induced on the following day (day 0). On day 18, CNS-infiltrated lymphocytes were collected, and a FACS analysis of Vα3.2 and Vβ11 expression in CD45.1- or CD45.2-positive CD4$^+$ T cells was performed. Representative FACS data are shown. **(b)** Cumulative data from five mice in two experiments. Each dot represents an individual recipient mouse.

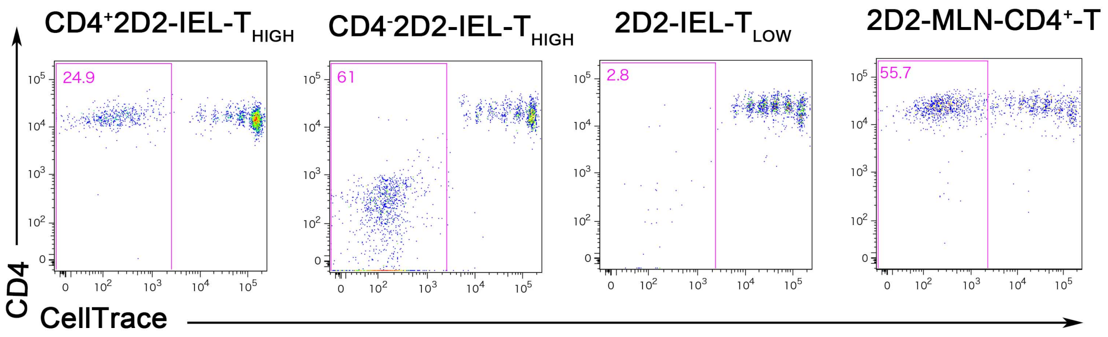

Supplementary Fig. 6: Detection of suppressor cells after co-culture with responder cells. After co-culture of CellTrace-labelled responder cells and CD4$^+$/ CD4$^-$2D2-IEL-T$_{HIGH}$, 2D2-IEL-T$_{LOW}$, or 2D2-MLN-CD4$^+$-T cells, as depicted in Fig. 6a, cells were analysed by FACS. 7AAD$^-$TCRβ$^+$ cells were gated, and the proportion of CellTrace-negative cells is displayed. Data are representative of three experiments.

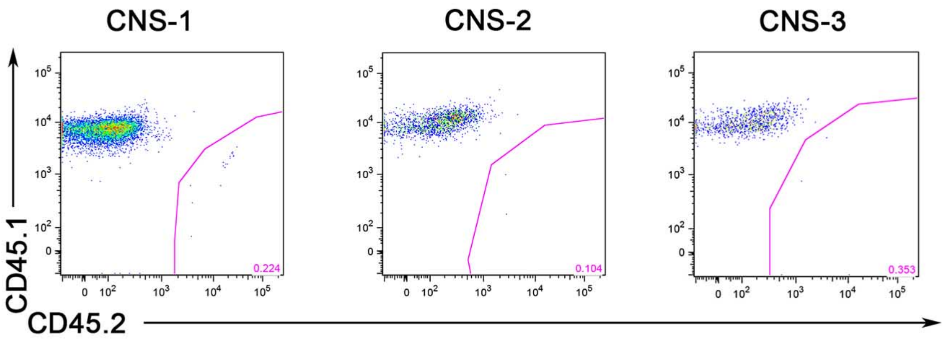

Supplementary Fig. 7: Transferred CD4+2D2-IEL-T_{HIGH} cells hardly infiltrate the CNS of naive recipient mice.

CNS lymphocytes from three samples (CNS1-3, results in Fig. 9) were gated for 7AAD-CD4$^+$ cells and were examined for the expression of CD45.1 and CD45.2.

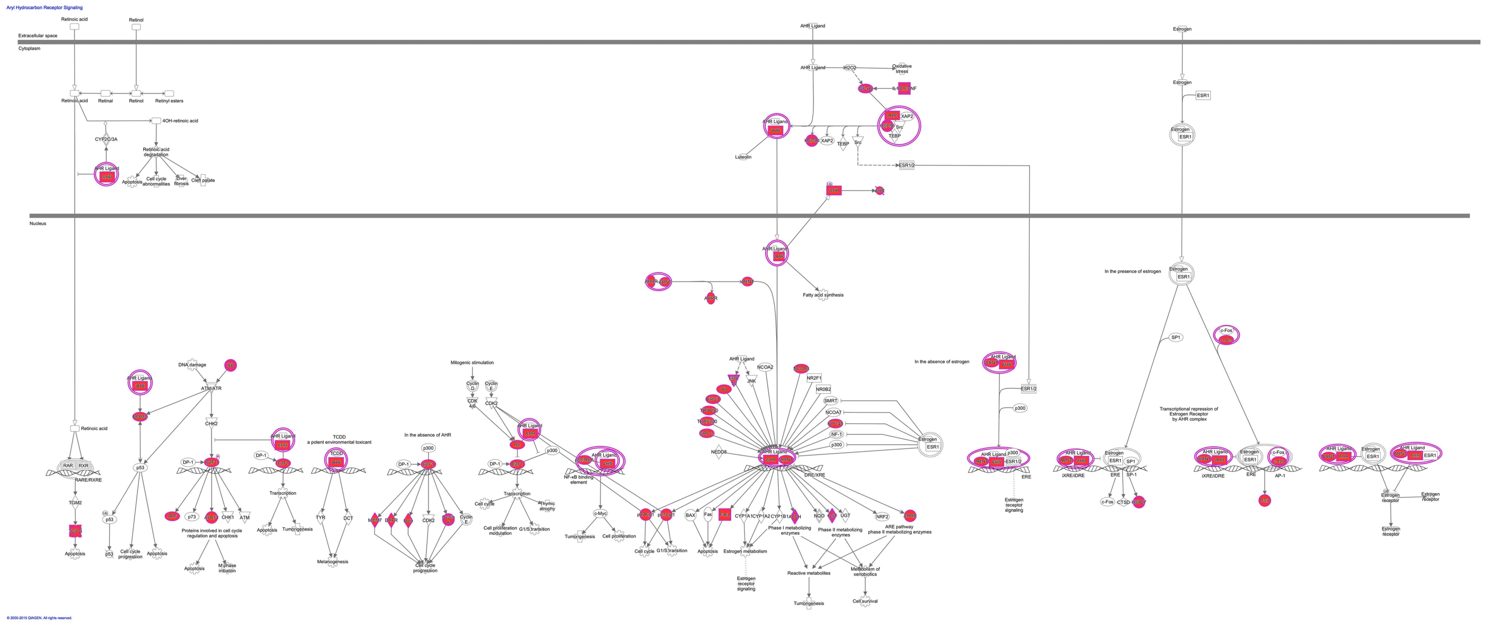

Supplementary Fig. 8: AHR signaling pathway-related genes were expressed more highly in CD4$^+$ IELs than in spleen CD4$^+$ T cells.

An illustration of the AHR signaling pathway obtained from IPA software is displayed. The genes selected in Fig. 10b are highlighted in red.

Gene	Forward primers	Reverse primers
Gapdh	5'-AACGACCCCTTCATTGAC-3'	5'-TCCACGACATACTCAGGAC-3'
Il17a	5'-GCGCTGTGTCAATGCGGAGG-3'	5'-CTGGCGGACAATCGAGGCCA-3'
Ifng	5'-AACCCACAGGTCCAGCGCCA -3'	5'-CCCCGAATCAGCAGCGACTCC-3'
Il10	5'-CATGGGTCTTGGGAAGAGAA-3'	5'-CATTCCCAGAGGAATTGCAT-3'
Tgfb1	5'-ACCATGCCAACTTCTGTCTG-3'	5'-CGGGTTGTGTTGGTTGTAGA-3'
Ccr5	5'-GCCAGAGGAGGTGAGACATCCGT-3'	5'-GGCAGGAGCTGAGCCGCAAT-3'
Cxcr3	5'-CCGCCCTGCCCACAGGATTTC-3'	5'-CGCCCCATTGCCTAGCAGCC-3'
Ccr9	5'-AGGCAGCTGCAGTGGTCCTCTC-3'	5'-ACCAGCCTCCAGTGGCAAAGG-3'
Ccr6	5'-CTTTGGGGAGCCCAGCTTGGAG-3'	5'-GGCCCTGGTCACAGGGGACTG-3'
Ccr2	5'-TCAGCTGCCTGCAAAGACCAGA -3'	5'-ACGGTGTGGTGGCCCCTTCAT-3'
Ccr7	5'-CGGAACGCGTCGGTGAGCAT-3'	5'-TGTAGTCCGGGCAGGGGAGC-3'
Ctla4	5'-TAGCAGCCATGGTGTCGCCAG-3'	5'-TCTGCCGCAGCACAGTCACC-3'
Rorc	5'-TCTACGGCAGCGCACCAACC-3'	5'-GGGCACAGCGCTCCCACATC-3'
Maf	5'-AGGATGGCTTCAGAACTGGC-3'	5'-GGTCTCCACCGGTTCCTTTT-3'
Pdcd1	5'-AACCAGAAGGCCGGTTTCAA-3'	5'-AGTGTCGTCCTTGCTTCCAG-3'
Pdl1	5'-CGCCTGCAGATAGTTCCCAA-3'	5'-ATCGTGACGTTGCTGCCATA-3'
Thpok	5'-ATGGGATTCCAATCAGGTCA-3'	5'-TTCTTCCTACACCCTGTGCC-3'
Runx3	5'-ACAGCATCTTTGACTCCTTCC-3'	5'-TGTTCTCGCCCATCTTGC-3'

Supplementary Table 1: The specific primers used for real-time PCR.
